# The herpes simplex origin-binding protein: mechanisms for sequence-specific DNA binding and dimerization revealed by Cryo-EM

**DOI:** 10.1093/nar/gkaf1029

**Published:** 2025-10-23

**Authors:** Emil Gustavsson, Kay Grünewald, Per Elias, B Martin Hällberg

**Affiliations:** Department of Cell and Molecular Biology, Karolinska Institutet, Stockholm 171 77, Sweden; CSSB Centre for Structural Systems Biology, Deutsches Elektronen-Synchrotron DESY, Notkestraße 85, Hamburg 22607, Germany; CSSB Centre for Structural Systems Biology, Deutsches Elektronen-Synchrotron DESY, Notkestraße 85, Hamburg 22607, Germany; Leibniz-Institute of Virology (LIV), Structural Cell Biology of Viruses, Martinistraße 52, Hamburg 20251, Germany; Department of Chemistry, University of Hamburg, Martin-Luther-King-Platz 6, Hamburg 20146, Germany; Institute of Biomedicine, Department of Medical Biochemistry and Cell Biology, Sahlgrenska Academy, University of Gothenburg, Box 440, Gothenburg 405 30, Sweden; Department of Cell and Molecular Biology, Karolinska Institutet, Stockholm 171 77, Sweden; CSSB Centre for Structural Systems Biology, Deutsches Elektronen-Synchrotron DESY, Notkestraße 85, Hamburg 22607, Germany

## Abstract

Herpes simplex viruses 1 and 2 (HSV-1,2) present growing treatment challenges due to increasing resistance to antivirals targeting viral DNA polymerase, particularly in immunocompromised individuals. The HSV-1 origin-binding protein (OBP), an essential Superfamily 2 (SF2) DNA helicase encoded by the UL9 gene, is a promising alternative therapeutic target. Here, we present cryo-EM structures of OBP at up to 2.8 Å resolution in multiple conformational states, including complexes with the OriS recognition sequence and the non-hydrolyzable ATP analog ATPγS. The structures reveal an unexpected head-to-tail dimer stabilized by the *C*-terminal domain, where the conserved RVKNL motif mediates sequence-specific DNA recognition. The *C*-terminal domain extends into the partner monomer, suggesting a regulatory mechanism involving the single-stranded DNA-binding protein ICP8. We also resolve an OBP monomer bound to a DNA hairpin with a 3′ single-stranded tail (mini-OriS*), and at lower resolution, a dimer-dimer assembly of two OBP dimers bound simultaneously to OriS or mini-OriS*. These structures uncover the molecular basis of HSV-1 origin recognition and unwinding, and identify multiple druggable interfaces, laying the groundwork for structure-based antiviral development targeting HSV-1 OBP.

## Graphical Abstract

**Graphical Abstract:**
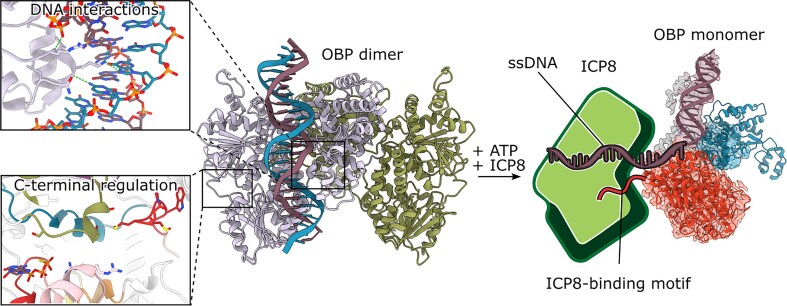

## Introduction

Herpes simplex virus 1 (HSV-1) infections present a growing challenge due to resistance to antiviral drugs that target viral DNA polymerase. This resistance is especially problematic for patients undergoing immunosuppressive therapy, in whom drug-resistant strains emerge much more frequently [1–3]. Since approximately 70% of the global population carries HSV-1 [4], with symptoms ranging from common cold sores to potentially fatal encephalitis, alternative therapeutic approaches are in demand.

**Figure 1.**
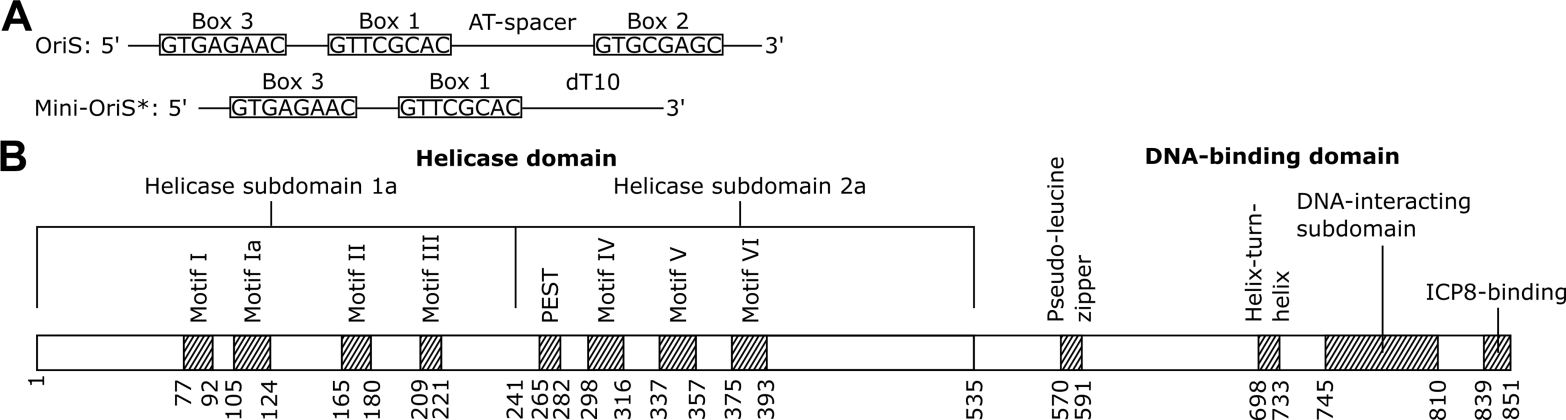
Schematic overview of OriS and OBP conserved regions. (**A**) OriS contains three binding sites for OBP, designated as Box 1, Box 2, and Box 3, with decreasing binding affinities. Box 2 is inversely oriented compared to Box 1, with an AT-rich spacer sequence in between. Also, Box 3 is inverted in relation to Box 1. In OriS*, Box 3 and Box 1 form a hairpin with a 12-nucleotide stem and a loop of three nucleotides [41]. A minimal version of OriS*, consisting of a hairpin and a single-stranded tail (mini-OriS*), binds tightly to OBP and efficiently stimulates OBP’s ATPase activity. (**B**) An overview of the conserved motifs of OBP. The *N*-terminal helicase domain is divided into two conserved subdomains (1a and 2a) containing seven signature motifs of SF1 and SF2 helicases. It also contains a proposed PEST motif. The *C*-terminal DNA-binding domain contains, in addition to the highly conserved RVKNL motif responsible for sequence-specific origin recognition, a pseudo-leucine zipper, a helix-turn-helix motif, and the ICP8-binding motif in the extreme *C*-terminus.

A detailed understanding of the HSV-1 genome organization and replication initiation is essential to unraveling the replication mechanism. The viral genome consists of a linear 152 kbp double-stranded DNA with two unique regions – Unique Long (UL) and Unique Short (US) – each flanked by inverted repeat sequences [5]. Upon infection, this linear genome circularizes in the nucleus to enable rolling-circle replication [6]. Viral DNA synthesis is initiated from three redundant origins of replication: OriL, located within the UL segment, and two copies of OriS in the inverted repeats flanking the US region [7–14]. OriS contains a sophisticated recognition system comprising three binding sites (Box 1, Box 2, and Box 3) for the origin-binding protein (OBP), with progressively decreasing binding affinity (Fig. 1A, Table 1) [10, 15]. All three sites are essential for DNA replication and ATP-dependent unwinding of OriS [16, 17]. Their spatial organization is critical for function: Boxes 1 and 2 are inversely oriented and separated by an AT-rich spacer sequence that facilitates cooperative binding [15, 18]. Box 3, also inverted relative to Box 1, serves a specialized role in forming a stable hairpin-loop structure in unwound OriS referred to as OriS* [17, 19, 20].

**Table 1.** Sequences for the OriS recognition sites

Recognition site	Sequence upper strand 5′-3′	Sequence lower strand 3′-5′
Box 1	**GTTCGCAC**	
Box 2	GTGCGAGC	**GCTCGCAC**
Box 3	GTGAGAAC	**GTTCTCAC**

HSV-1 gene expression follows a tightly regulated cascade during lytic infection: immediate early (IE) genes are expressed first, followed by early (E) and late (L) genes [21]. The early genes encode seven essential replication proteins, including the DNA polymerase complex, helicase–primase complex, single-stranded DNA binding protein ICP8, and the OBP [22]. These proteins interact in a coordinated manner to drive the initiation and progression of DNA synthesis [23, 24].

At the core of HSV-1 replication initiation is the OBP, a 94 kDa protein encoded by the UL9 gene [7, 25]. As an essential component of the viral replication machinery [22, 26], OBP integrates multiple critical functions: origin recognition, DNA binding, and ATP-dependent helicase activity (Fig. 1B) [27–29]. OBP can unwind 200 bp of DNA alone, but in the presence of the viral single-stranded DNA binding protein ICP8, its unwinding capacity extends to 2 kb [27, 30–32]. In solution, OBP predominantly forms homodimers.

OBP interacts with the double-stranded OriS replication origin by forming two distinct complexes: complex I and complex II. DNase I footprinting and gel retardation assays reveal that one OBP dimer binds to Box 1 in complex I, while in complex II, two OBP dimers cooperatively bind to both Box 1 and Box 2. The AT-rich spacer between Boxes 1 and 2 influences complex II formation, as a six-base-pair deletion in this region abolishes replication but enhances complex II formation [18]. OBP also binds to the hairpin-loop structure in OriS*, which forms during replication initiation [19]. Exonuclease I digestion experiments suggest that OBP’s *C*-terminal domain binds to the hairpin while its *N*-terminal helicase domain interacts with the 3′ single-stranded tail [33]. A minimal OriS* (mini-OriS*) containing a Box 3-Box 1 hairpin and a 3′ single-stranded tail of at least 10 nucleotides efficiently stimulates OBP’s ATPase activity (Fig. 1A). Co-precipitation experiments further indicate that an OBP dimer can bind two Box 1 duplexes. Under identical experimental conditions, only a single OriS* hairpin with a single-stranded tail is bound [33]. It is therefore likely that exposure to single-stranded DNA significantly alters OBP’s structural and biochemical properties, underscoring its dynamic role in replication initiation.

OBP’s diverse functions are enabled by its modular architecture, characteristic of superfamily 2 (SF2) DNA helicases. SF2 helicases participate in both DNA and RNA metabolism and replication, with viral members playing essential roles in nucleic acid unwinding [34]. While bacterial and nuclear eukaryotic replication initiators are AAA + hexameric helicases [35, 36], OBP represents a distinct strategy for origin recognition and activation.

OBP consists of two major functional domains: an *N*-terminal helicase-like domain (residues 1–535) and a *C*-terminal origin-binding domain (residues 536–851). The helicase domain contains two conserved subdomains, 1a (residues 1–241) and 2a (residues 242–535) [23, 37, 38], and contains seven signature motifs shared across SF1 and SF2 helicases (Fig. 1B). A truncated version of OBP (residues 1–535) retains ATPase and helicase activities, consistent with its SF2 classification [37].

The *C*-terminal DNA-binding domain exists as a monomer in solution and binds with the highest affinity to Box 1, interacting with both the major groove and phosphodiester backbone [15, 39, 40]. This domain contains a highly conserved R756VKNL motif, which is found across alphaherpesviruses [41] and provides sequence-specific recognition of viral origin sequences [29].

Additionally, OBP interacts with ICP8 through a conserved 13-residue motif in the extreme *C*-terminus of OBP (W839PXXXGAXXFXX(L/I)), significantly enhancing helicase activity [41]. This interaction does not alter OBP’s intrinsic ATPase, and it is suggested that it may modulate the coupling between ATP hydrolysis and helicase activity [42]. Notably, the OBP–ICP8 complex forms in the absence of a DNA substrate [32, 43].

Despite OBP’s identification four decades ago [7] and extensive biochemical and mutational studies [15, 17, 18, 20, 32, 33, 40, 41, 43–46], its three-dimensional structure has remained elusive. This knowledge gap is particularly significant not only for our understanding of the mechanisms of HSV-1 replication but also for therapeutic development. The current anti-HSV-1 treatments exclusively target the viral DNA polymerase complex, but the emergence of resistant strains underscores the need for alternative strategies. OBP represents an especially promising antiviral target because it acts at the earliest stages of viral DNA replication, prior to polymerase recruitment. Structural insights into OBP could thus serve dual purposes: uncovering the fundamental mechanisms of viral DNA replication and providing a foundation for the development of novel antivirals targeting this essential and previously unexploited step in the viral life cycle.

We used electron cryo-microscopy (cryo-EM) to determine the first high-resolution structures of OBP in multiple functional states, offering unprecedented insights into the initiation of HSV-1 DNA replication. Our structures capture key snapshots of the process, including OBP bound to its OriS recognition sequence and complexed with a non-hydrolyzable ATP analog. Additionally, we observed OBP in various oligomeric states, ranging from monomers to dimer-dimer assemblies, revealing unexpected assembly mechanisms. These structures elucidate the molecular basis of origin recognition and highlight the dramatic conformational changes that accompany OBP activation. By visualizing OBP’s interactions with both DNA and ATP, we provide new mechanistic insights into viral origin activation and identify multiple potential druggable sites. Our results pave the way for novel strategies for antiviral drug design that could bypass existing resistance mechanisms and perhaps prevent reactivation from latency.

## Materials and methods

### Construction of expression vectors

The HSV-1 UL9 gene (gene-ID: 2 703 434) was codon optimized for expression in insect cells and ordered as gene fragments with an *N*-terminal Strep-tag from GenScript. NEBuilder HiFi DNA Assembly Cloning Kit (NEB, cat. #E5520S) was used to assemble the gene fragments into a pFastBac plasmid without additional affinity tags (Gibco, cat. #10360014). The sequence-verified pFastBac plasmid containing gene UL9 was transformed into DH10Bac cells (Gibco, cat. #10361012), and bacmid incorporation was analyzed with two rounds of blue-white screening and PCR of resulting bacmids.

### Protein expression and purification

The Gibco ExpiSf Expression System (cat. #A38841) from Thermo Fisher Scientific was used for the development of recombinant baculovirus and protein expression. ExpiSf9 cells were transfected with recombinant bacmid, and P0 baculovirus stock was harvested after 96 h of transfection. Subsequently, the P0 baculovirus stock was used to infect ExpiSf9 cells for expression of OBP. Cells were harvested after 48 h and lysed in lysis buffer (20 mM HEPES pH 7.8, 400 mM NaCl, 1.5 mM MgCl2, 1 mM DTT, 5% glycerol (v/v), 1x cOmplete protease inhibitor cocktail (Roche, cat. #11 697 498 001), 1 μg/ml DNase I (ITW reagents, cat. #A3778) and 0.1% (v/v) Triton X-100 (ITW reagents, cat. #142 314)) with a Dounce homogenizer. Cell debris was spun down in two rounds of centrifugation (20 min at 15 000*g followed by 40 min at 55 000*g), and the supernatant was filtered through a 0.2 μm syringe filter. The supernatant was then loaded onto a StrepTrap HP column (Cytiva, cat. #28 907 547) equilibrated with equilibration buffer (20 mM HEPES pH 7.8, 400 mM NaCl, 1.5 mM MgCl2, 1 mM DTT, 5% glycerol (v/v)). Protein was eluted by adding 10 mM desthiobiotin (IBA Life Sciences, cat. #2–1000–002). Protein purity was analyzed with SDS-PAGE.

### *In vitro* reconstitution of protein-DNA complexes for cryo-EM

In general, the *in vitro* reconstitution of all samples followed the same procedure, with differences noted below. All DNA oligonucleotides were ordered from Integrated DNA Technologies. Protein buffer was exchanged to 20 mM HEPES pH 7.8, 150 mM NaCl, 5 mM MgCl_2_, 2 mM DTT, and 5% glycerol, and the protein was concentrated to 2 μM in a Vivaspin 500 centrifugal concentrator with a 50 000 MWCO cutoff (Sartorius, cat. #VS0102). DNA oligos were added in a protein:DNA molar ratio of 1:1.2. The reaction was incubated at 37°C for 30 min and aliquots of 2.5 μl were then applied to glow-discharged UltrAufoil R1.2/1.3 Au 300 mesh grids (Quantifoil), immediately blotted for 5 s and plunged into liquid ethane using a Vitrobot IV (Thermo Fisher) at 4°C and 95% relative humidity.

*Dimeric OBP bound to OriS-6AT oligomer*. OriS-6AT DNA sequence: 5′–AAA AGA AGT GAG AAC GCG AAG CGT TCG CAC TTC GTC CCA ATA TAT TAT TAG GGC GAA GTG CGA GCA CTG GCG CC–3′

*Dimeric OBP bound to OriS oligomer and ATPγS*. OriS DNA sequence: 5′–AAA AGA AGT GAG AAC GCG AAG CGT TCG CAC TTC GTC CCA ATA TAT ATA TAT TAT TAG GGC GAA GTG CGA GCA CTG GCG CC–3′

OBP bound to mini-OriS*. Mini-OriS* sequence: 5′–AAA AGA AGT GAG AAC GCG AAG CGT TCG CAC TTC GTT TTT TTT TT–3′

The hairpin is composed of a 12-nucleotide stem with one mismatch and three-nucleotide loop as determined by NMR, and it has a melting point of 71°C [41].

### Cryo-EM grid preparation and data collection

Data collection of the OBP + OriS-6AT, OBP + OriS + ATPγS, and OBP + mini-OriS* dimer-dimer assembly datasets. A Krios G3i electron microscope (Thermo Fisher) at the Centre for Structural Systems Biology (CSSB) Cryo-EM facility, operated at an accelerating voltage of 300 kV, equipped with a K3 BioQuantum (Gatan) was used for collecting the OBP + OriS-6AT, OBP + OriS + ATPγS, and OBP + mini-OriS* datasets. Cryo-EM data were acquired using EPU software (Thermo Fisher) at a nominal magnification of 105 kX, with a pixel size of 0.85 Å per pixel. Movies of a total fluence of 50 (OBP + OriS-6AT and OBP + mini-OriS* *dimer-dimer assembly*) or 54 (OBP + OriS + ATPγS) e^−^/Å^2^ were collected at 1 e^−^/Å^2^ per frame. A total of 4021 (OBP + OriS-6AT), 5554 (OBP + OriS + ATPγS), and 3864 movies were acquired at an underfocus range of 0.75 to 2.0 μm.

OBP + mini-OriS* monomer dataset. A Krios G3i electron microscope at the Karolinska Institutet (KI) 3D-EM facility, operated at an accelerating voltage of 300 kV, equipped with X-FEG, and a K3 BioQuantum (Gatan) was used for collecting OBP + mini-OriS* monomer data. Cryo-EM data were acquired using EPU software (Thermo Fisher) at a nominal magnification of 165 kX, with a pixel size of 0.505 Å per pixel. Movies of a total fluence of 58 e^−^/Å^2^ were collected at 0.69 e^−^/Å^2^ per frame. A total of 24,100 movies were acquired at an underfocus range of 0.5–2.0 μm with a tilted stage of 20°.

### Cryo-EM data processing

Motion correction was performed with RELION’s own implementation of MotionCor2 [47] and CTF estimation with CTFFind 4.1 [48]. Particles were picked using Warp 1.0.9 [49] and extracted in RELION 4.0b [50]. The particles were then imported to cryoSPARC v.3.2.0 [51]. One round of 2D classification was performed for each dataset, and the subsequent specific refinement strategies for each dataset are described below.

*OBP + OriS-6AT and OBP + OriS + ATPγS datasets*. Following 2D classification, ab-initio reconstruction was performed with 5 or 4 classes for OBP + OriS-6AT and OBP + OriS + ATPγS, respectively. Two rounds of heterogeneous refinement were performed. This was followed by two (OBP + OriS-6AT) or three (OBP + OriS + ATPγS) more rounds of heterogeneous refinement, including all picked particles to recover good particles initially excluded in the 2D classification, together with an additional “noise class” reconstructed from 10 000 particles excluded during an initial 2D classification. A non-uniform refinement was performed on the best class. The resulting particles were exported to RELION for Bayesian polishing and CTF refinement. The particles were then re-imported into cryoSPARC for one final non-uniform refinement, followed by local refinement focusing on monomers A and B, respectively. Representative processing workflows for OBP + OriS-6AT and OBP + OriS + ATPγS can be seen in Supplementary Figs S1 and S2, respectively.

OBP + mini-OriS* monomer dataset. After 2D classification, ab-initio reconstruction with five classes was conducted, followed by heterogeneous refinement with one additional “noise class” reconstructed from 10 000 particles excluded during the initial 2D classification. Additional separate heterogeneous refinements were performed on the two most prominent classes, followed by non-uniform refinements. These two classes were combined into a new round of heterogeneous refinement, with 3 additional “noise classes.” These volumes, together with all picked particles, were used in 7 new rounds of heterogeneous refinement, followed by a new non-uniform refinement. Three rounds of rebalance orientations and non-uniform refinement were used to minimize preferred-orientation problems. A representative workflow can be seen in Supplementary Fig. S3.

OBP + mini-OriS* dimer-dimer assembly dataset. After 2D classification, *ab initio* reconstruction with three classes was conducted, followed by heterogeneous refinement with two additional “noise classes” reconstructed from 10 000 particles excluded during the initial 2D classification and a non-uniform refinement. Two rounds of heterogeneous refinement were performed, including all picked particles, the non-uniform refinement volume, and three additional “noise classes.” Finally, a non-uniform refinement was performed, followed by local refinement on dimers 1 and 2, respectively. A representative workflow can be seen in Supplementary Fig. S4.

### Cryo-EM post-processing and model building

The half maps from the local refinement jobs were merged in ChimeraX v.1.3 [52] and post-processed with LocSpiral [53], DeepEMhancer [54], or by local resolution estimation and local filtering in cryoSPARC [51]. The initial model building involved fitting the AlphaFold2-predicted [55] helicase domain into the map for the OBP + OriS–6AT complex using Coot v.0.9.8 [56] combined with a model generated by ModelAngelo [57]. Iterative model building was performed in Coot, followed by Ramachandran and rotamer fitting in ISOLDE v.1.3 [58] and refinement with Servalcat [59]. Data collection parameters, refinement, and validation statistics can be found in Supplementary Table S1.

## Results

### Head-to-tail dimer architecture reveals novel regulatory interfaces

Our cryo-EM analysis provides the first structures of HSV-1 OBP, capturing two distinct early states during activation of OriS at resolutions of 2.8 Å and 3.5 Å, respectively (Supplementary Figs S5 and S6). The first structure, representing complex I, shows OBP bound to modified OriS, where the removal of six base pairs from the AT-rich region stabilizes cooperative binding of OBP to OriS but prevents unwinding initiation [18]. The second structure captures OBP bound to the complete OriS recognition site, in complex with a non-hydrolyzable ATP analog (ATPγS).

**Figure 2.**
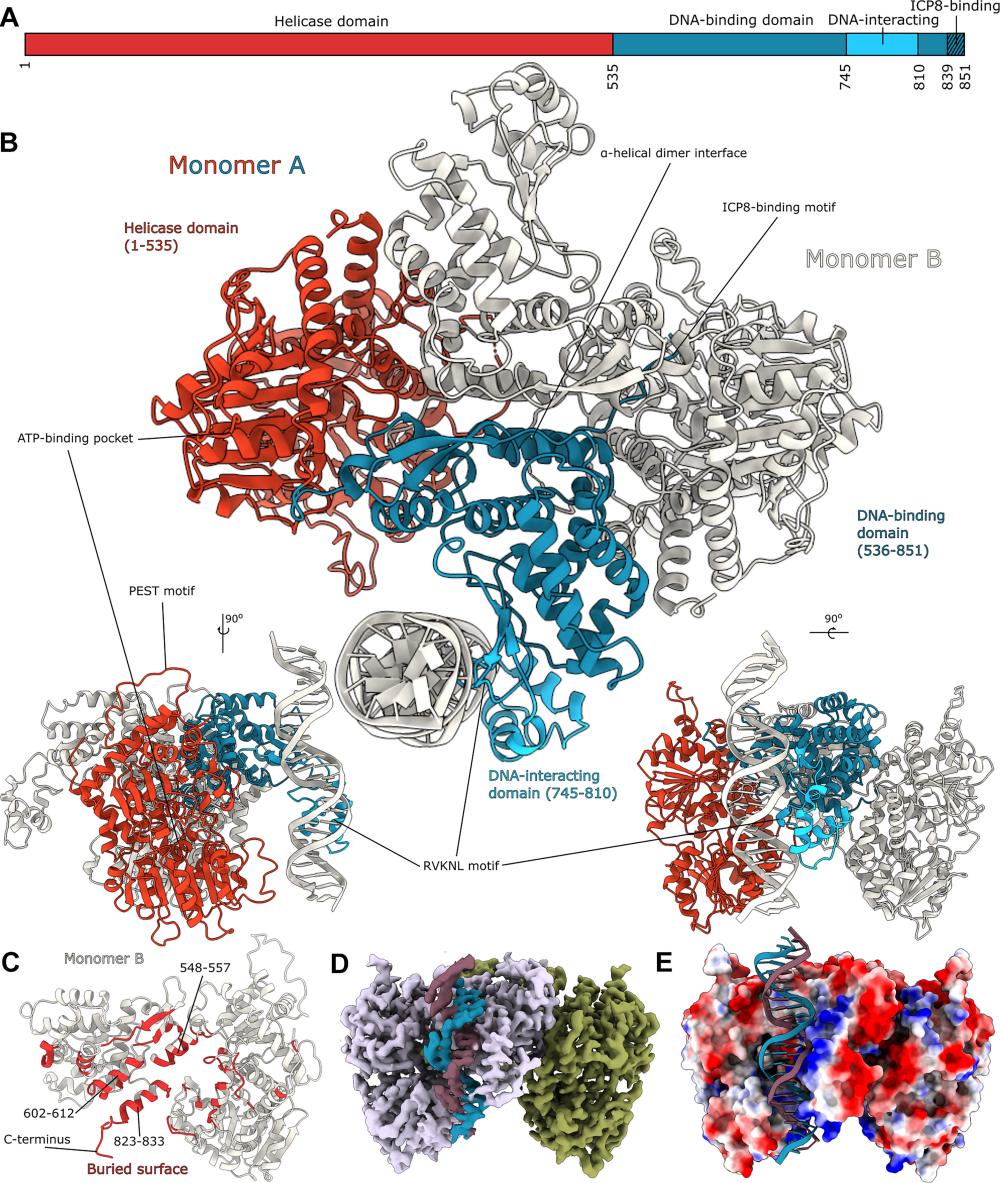
Structural overview of the HSV-1 OBP. (**A**) Linear domain architecture of the OBP. (**B**) Dimer of the OBP, corresponding to complex I [18]. Monomer A (red and blue) binds a double-stranded oligomer of OriS-6AT (white). Monomer B (white) without bound DNA. This arrangement differs from previous suggestions of primary contacts between *N*-terminal residues 292–321 and the *C*-terminus [60]. Organization of the *N*-terminal helicase domain (red, residues 1 to 535) and the *C*-terminal DNA-binding domains (blue, residues 536–851) in monomer A. (**C**) An analysis of the buried surface between monomers shows the dimer interface (red) in monomer B (white). The interface is mainly made up of three central α-helices in the *C*-terminal domain, as well as the extreme *C*-terminus. (**D**) Cryo-EM map colored plum (monomer A), green (monomer B), blue, and purple (DNA). (**E**) A molecular surface representation of the OBP dimer with bound DNA, colored by the local electrostatic potential (blue, +8 kT; red − 5 kT). A distinct positively charged DNA-binding surface can be identified, while the remaining surface is negatively charged.

The structures reveal a head-to-tail dimer architecture, with DNA binding on one side. Each monomer consists of two major domains: an *N*-terminal helicase domain (residues 1–535) and a *C*-terminal DNA-binding domain (residues 536–851) (Fig. 2A and B). The primary dimer interface is formed by three α-helices in the *C*-terminal domain (residues 548–557, 602–612, and 823–833), along with additional contacts from loops and a β-strand (Fig. 2C, red). This arrangement challenges previous models, suggesting that primary contacts occur between *N*-terminal residues 292–321 and the *C*-terminus [60]. Notably, the extreme *C*-terminus of each monomer extends into its partner’s *N*-terminal domain, forming an extended network of inter-monomer contacts that may regulate protein function.

The two structures described above correspond to complex I with one dimer of OBP bound to the high-affinity site Box 1 (Fig. 2B and D) as described by Gustafsson *et al.* [18]. Cooperative binding of OBP to Boxes 1 and 2 would require two dimers to form a dimer-dimer assembly (see below). This arrangement implies that substantial conformational changes occur in OriS between initial DNA recognition and the active unwinding state, possibly accompanied by conformational changes also in OBP.

Electrostatic surface analysis reveals a striking charge distribution, with a distinctly positive DNA-binding surface surrounded by predominantly negative charges across the rest of the protein (Fig. 2E). This charge segregation likely aids in DNA binding orientation and may contribute to the specificity and stability of complexes between OBP and OriS.

Beyond these dimeric structures, we also captured OBP in two additional functionally relevant states: as a monomer bound to mini-OriS* and as a dimer-dimer assembly composed of two dimers. These alternate states may provide valuable insights into the protein's conformational flexibility during DNA replication initiation.

### Conserved motifs in the ATP-binding pocket and their interactions with the OBP *C*-terminus hint at a regulatory mechanism

**Figure 3.**
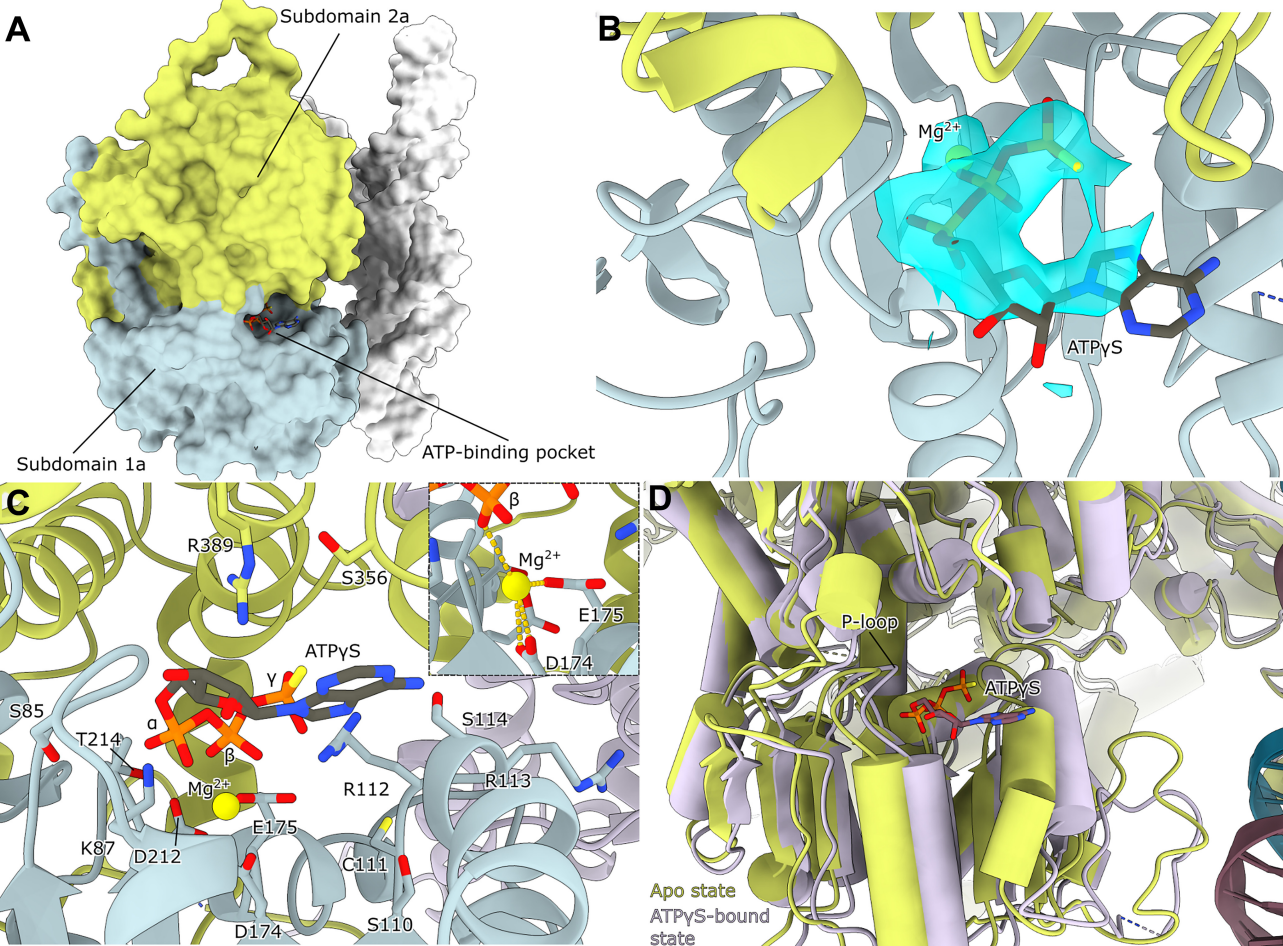
Arrangement of the OBP helicase domain (**A**) Surface view of the OBP + OriS + ATPγS structure. The conserved helicase subdomains 1a and 2a are colored light blue and light green, respectively. (**B**) Sharpened refinement map around the ATPγS and metal ion reveals an unusual positioning. Model colored as in A. (**C**) A number of conserved residues in the ATP-binding pocket are shown, and the catalytic Mg^2+^ is colored in yellow. Metal coordination can be seen in the inset. The metal ion is coordinated by D174 and E175; other coordinating residues were not identified. The distance to the β-phosphate is 3 Å. Model colored as in A. (**D**) Overlay of ATPγS-bound (plum) and unbound (light green) states shows the pocket closing slightly around the ATPγS. The P-loop in motif I shifts closer to the ATPγS.

The cryo-EM structures reveal the detailed organization of the OBP helicase domain, which consists of subdomains 1a and 2a that form a central ATP-binding pocket (Fig. 3A). In our OBP + OriS + ATPγS complex, we observe both the ATP analog and a coordinated Mg^2+^ ion (Fig. 3B and C), capturing the nucleotide-bound state. ATPγS binding induces subtle closure of the pocket's lower half and a shift of the motif I P-loop, suggesting conformational changes that may couple ATP hydrolysis to DNA unwinding (Fig. 3D).

**Figure 4.**
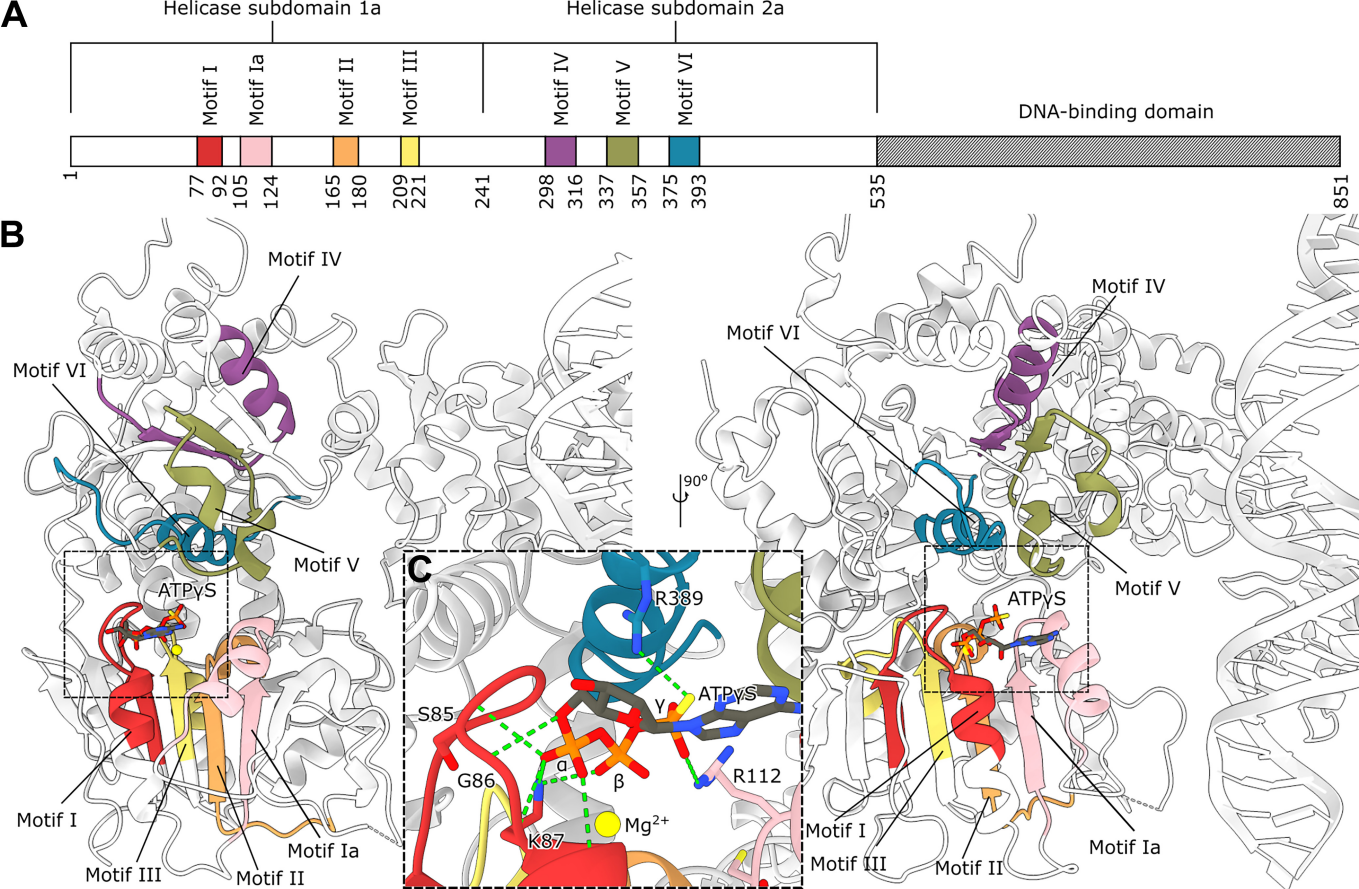
(**A**) Linear domain architecture of the helicase domain. (**B**) The conserved motifs in Superfamily 1 and 2 helicases are colored as follows: Motif I (red), Motif Ia (salmon), Motif II (orange), motif III (yellow), motif IV (purple), motif V (green), motif VI (blue). (**C**) OBP interacts with ATPγS through hydrogen bonds between S85, G86, K87, and T88 backbone amides and the α-phosphate. K87 in motif I also interacts with the β-phosphate. In addition, both R389 and R112 hydrogen bond to the γ-phosphate.

The ATP-binding pocket is defined by seven conserved motifs characteristic of SF1/SF2 helicases (Fig. 4A,B): motifs I (residues 77–92), Ia (105–124), II (165–180), III (209–221), IV (298–316), V (337–357), and VI (375–393) [61]. Within this pocket, we observe a seemingly unusual nucleotide interaction pattern: K87 of motif I contacts the α- and β-phosphates of ATPγS, while backbone amides from S85, G86, K87, and T88 form hydrogen bonds with the α-phosphate (Fig. 4C). This arrangement differs from other helicases, where the equivalent conserved lysine typically contacts the β- and γ-phosphates [62, 63]. The ATPγS seems to have settled further into the pocket, and although it cannot be fully ruled out that ATPγS adopts an unnatural conformation in OBP, structures of other ATPγS-bound helicases show the typical conformation [64–68]. Previous mutational studies of the ATP-binding pocket have not distinguished OBP from other helicases [61, 69]. Therefore, our structure potentially represents a non-catalytic state before ATP adopts a position in its pocket that is consistent with turnover.

Within motif Ia, our structure shows that R112 reorients to interact with the γ-phosphate of ATPγS. This provides a structural basis for previous biochemical findings that R112A and R113A mutations selectively abolish ssDNA binding while preserving ATPase activity [69]. Comparison with the hepatitis C virus nonstructural protein 3 (NS3) RNA helicase structure, the most well-studied SF2 helicase (Supplementary Fig. S7) [70] further supports the role of these residues in engaging the phosphate backbone during DNA unwinding. Notably, S110 occupies a strategic position between the catalytic site and these arginines, explaining its essential role in coupling ATPase and helicase activities [69]. Nearby residues C111 [71] and S114 may also contribute to this energy-coupling mechanism, suggesting a coordinated network of interactions that drives DNA unwinding.

The catalytic Mg^2+^ ion is coordinated by D174 and E175 within motif II (Fig. 3C inset), underscoring their critical role in helicase activity, as evidenced by the loss of function in E175A mutants [72]. Intriguingly, motif III’s D212 faces away from the ATP pocket, suggesting a structural rather than catalytic role—a finding consistent with the effects of nearby A213 and T214 mutations on helicase activity [72]. Similarly, motif IV’s position suggests a potential ssDNA-binding role during unwinding, as inferred from NS3 comparisons [70]. These observations reveal how distinct motifs contribute to different aspects of OBP’s helicase function.

### Dimer-partner *C*-terminal threading as a regulatory switch for helicase function

The structure also reveals important features beyond the immediate ATP-binding site. In motif V, we identify a critical loop containing the conserved G354, which explains why mutations at this position destabilize the entire protein [72]. This loop also positions S356 to face the ATP pocket. Motif VI exhibits an unexpected arrangement where R389 directly contacts the γ-phosphate of ATPγS (Fig. 4C), while R387, despite its conservation, remains too distant for ATP interaction. This configuration differs notably from the NS3 helicase [70], suggesting that OBP may employ a distinct regulatory mechanism for coupling ATP hydrolysis to DNA unwinding while maintaining essential nucleotide coordination functions.

**Figure 5.**
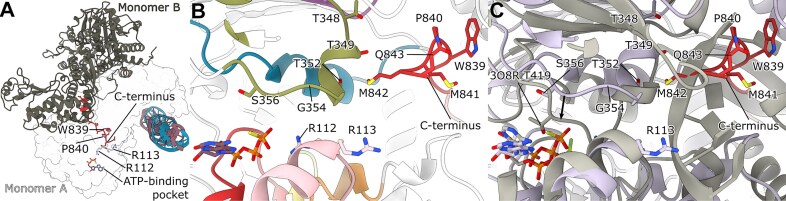
Arrangement of the OBP helicase domain and motifs. (**A**) The *C*-terminus (red) of monomer B (gray) stretches through monomer A (surface view) in close proximity to the ATP-binding pocket. Conserved residues R112 and R113 in monomer A, W839 and P840 in monomer B, as well as the *C*-terminal end, are also marked. (**B**) The conserved helicase motifs are colored as in Fig. 4B. The *C*-terminus of monomer B (red) stretches through monomer A in proximity to helicase motif V (green). A number of conserved threonines (T348, T349, and T352) interact with the *C*-terminus of the partner protomer. The conserved arginines in motif Ia (salmon) are located in close proximity to the *C*-terminus. The conserved residues W839 and P840, which are involved in interactions with ICP8, do not seem to have any direct interactions with residues in monomer A. (**C**) However, M842 pushes motif V out of position as compared to another SF2 helicase: NS3 (gray, PDB-ID: 3O8R) [73]. In NS3, T419 is in a position to interact with ATP, while S356 in OBP is out of position.

We also observe an unexpected feature of the extreme *C*-terminus, possibly explaining the unusual conformation of the helicase motifs described above. Each monomer threads its *C*-terminal end through its partner molecule, bringing it into close proximity with the ATP-binding pocket in the other monomer (Fig. 5A). This arrangement places the *C*-termini near the conserved arginines R112 and R113 of helicase motif Ia and motif V (Fig. 3C and 5B). Additionally, M842 pushes motif V out of position compared to other SF2 helicases, such as the hepatitis C virus NS3 (Fig. 5C, gray, PDB-ID: 3O8R) [73]. In NS3, T419 is positioned to interact with ATP, while the corresponding residue in OBP S356 is not. It is tempting to speculate that when the cross-protomer *C*-terminus is released, the helicase motifs enter their canonical positions around the ATP molecule, and that this would trigger ATP turnover.

Several conserved threonines (T348, T349, and T352) interact with the *C*-terminus. These structural observations provide a molecular explanation for previously puzzling biochemical data: C-terminal deletion mutants (lacking 27 residues) maintain origin-specific binding and exhibit enhanced helicase activity (8-fold increase) despite reduced DNA replication rates [44]. The positioning of the *C*-terminus may serve as an intrinsic regulatory element modulating both helicase activity and replication initiation.

Our structure also reveals the *N*-terminal portion of the ICP8-binding motif (WPXXXGAXXFXX(L/I) [41], with a clear map for the critical W839 and P840 residues along with three non-conserved amino acids (Fig. 5B). However, the *C*-terminal portion of this motif appears disordered in our structures. Compared to NS3, it seems the *C*-terminus threads through the potential ssDNA channel (Fig. 5C, Supplementary Fig. S7). The threading of the *C*-terminus through the partner monomer has important implications for ICP8 recruitment. Since the ICP8-binding motif is partially buried, a substantial structural reorganization must occur in response to ICP8 binding. Recruitment of ICP8 may thus induce conformational changes mechanistically linked to unwinding of OriS.

### Molecular basis of sequence-specific origin recognition

**Figure 6.**
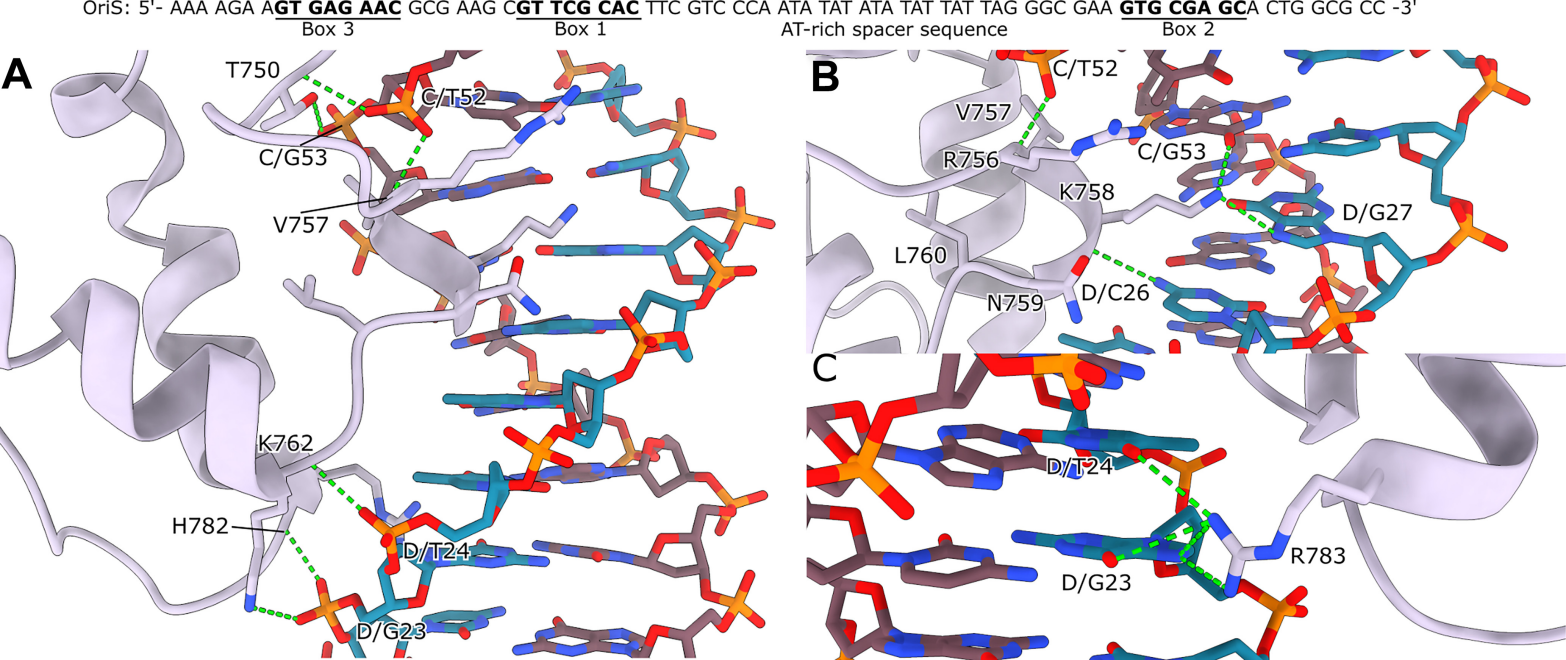
Conserved OBP–DNA interactions and residues important for sequence specificity. (**A**) The strongest interactions between OBP and the OriS-DNA (chain C, brown; chain D, blue) is made through D/G23, D/T24, and C/T52 [40]. The residues responsible for this interaction are: T750, which interacts with C/G53 and through the main chain amide with C/T52 phosphate group; V757 main chain amide also hydrogen bonds with the C/T52 phosphate. K762 interacts with both the phosphate group of D/G23 and through the main chain amide with D/T24 phosphate. H782 main chain amide interacts with D/G23 phosphate. (**B**) The conserved DNA-binding motif R756VKNL interacts with the DNA through sequence-specific interactions consisting of K758, which interacts with the D/C26 cytosine amine group through the main chain carbonyl. K758 also reaches into the major groove of the DNA and hydrogen bonds with the D/G27 guanine N7 and C/G53 guanine carbonyl group. These interactions would account for the discrimination between Boxes 1 and 3, as D/G27 is replaced by thymine in Box 3. In addition, V757 main chain amide hydrogen bonds to the phosphate group in C/T52. (**C**) Furthermore, R783 interacts with the D/G23 guanine carbonyl group and N7, as well as D/T24 thymine carbonyl group. The interaction with D/T24 would account for the discrimination between Boxes 1 and 2 (where the thymine is exchanged for a cytosine).

The *C*-terminal DNA-binding domain is divided into three subdomains (Fig. 2): (i) a helicase-proximal subdomain, which is required for proper folding, interacts with the helicase domains and contributes to formation of a stable OBP dimer, (ii) a DNA-interacting subdomain (residues 745–810) that recognizes the origin sequence, and (iii) a *C*-terminal extension with an α-helix (residues 823–833) that participates in dimer formation and, in addition, an ICP8 binding motif at the very end that also reaches over the dimer interface and interacts with the ATP binding site (see above). Our high-resolution OBP + OriS-6AT structure captures detailed interactions between the conserved R756VKNL motif and its target Box 1 sequence (GTTCGCAC) in the DNA major groove (Fig. 6A).

We observe two distinct types of protein-DNA contacts: sequence-specific base recognition and phosphate backbone interactions. The backbone contacts involve a network of main chain amides: H782 bonds with the D/G23 phosphate, K762 engages the D/T24 phosphate, and both T750 and V757 contact the C/T52 phosphate. This extensive interaction network aligns with previously identified contact points from biochemical studies [40], providing structural validation of earlier findings.

Within the RVKNL motif, K758 plays a critical role in sequence specificity through multiple contacts (Fig. 6B). This key residue makes both backbone and base-specific interactions: its main chain carbonyl recognizes the D/C26 cytosine's amine group, while its side chain reaches deep into the major groove to form hydrogen bonds with both C/G53 guanine's carbonyl and D/G27 guanine's N7. These interactions explain two fundamental aspects of origin recognition: the absolute conservation of C/G53 and D/C26 positions across all OriS boxes and OBP’s ability to discriminate between binding sites. Specifically, substituting D/G27 with thymine in Box 3 would create a steric clash with K758, offering the first structural explanation for how OBP differentially recognizes Box 1 versus Box 3 [15].

Within the major groove, we observe R756 near the invariant C/G53 and C/T52 bases. Although direct hydrogen bonds are not resolved in our structure, this positioning suggests a role in sequence recognition. Additional sequence-specific contacts are mediated by R783 (Fig. 6C), which forms an intricate network of interactions with D/G23 (both carbonyl and N7 positions) and D/T24’s carbonyl group. This precise arrangement explains another aspect of binding site selectivity: substituting D/T24 with cytosine in Box 2 would disrupt these R783-mediated interactions, providing a structural basis for the differential binding affinity between Boxes 1 and 2 [10].

Beyond the core recognition sequence, our structure reveals an unexpectedly extensive network of protein-DNA contacts (Supplementary Fig. S8). We identify multiple basic residues (K793, K802, R810, K746) as well as Q743, Y786, and potentially K780, engaging the DNA phosphate backbone through an elaborate hydrogen bonding network. This observation presents an interesting paradox: while previous biochemical studies indicated that sequence changes outside the core recognition site do not affect binding affinity [15], the extensive nature of these backbone contacts suggests they may play a more significant role in complex stability than previously appreciated. This apparent discrepancy between structural and biochemical observations raises important questions about the relationship between binding specificity and stability, warranting further investigation into the energetic contributions of these peripheral interactions.

### Structural insights redefine the roles of conserved motifs

**Figure 7.**
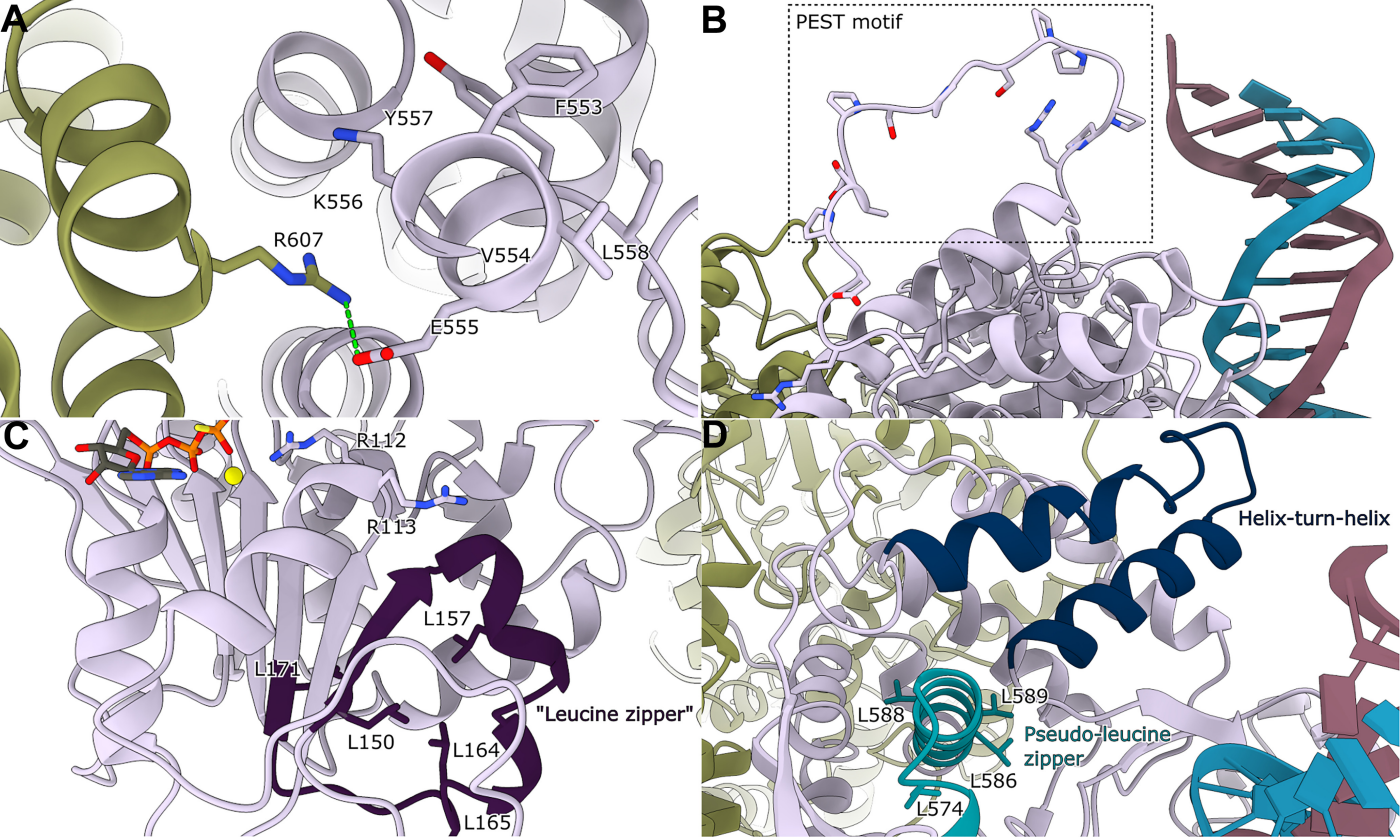
Conserved FXXKYL and PEST motifs. (**A**) The conserved motif F553XXKYL has been shown to be important for the correct folding of the DNA-binding domain [41], and a K556A mutation also causes temperature-sensitive OBP [74]. E555 in monomer A (plum) forms a hydrogen bond with R607 in monomer B (green). The conserved motif seems to play a structural role primarily. (**B**) The OBP contains a PEST motif (residues 265 to 282), which has been implicated in targeting proteins for ubiquitination and subsequent degradation [75, 76]. The PEST motif is located in an exposed, flexible loop at the apex of the protein structure. (**C**) Marked in purple is the proposed leucine zipper (residues 150–171) which do not form. (**D**) A pseudo-leucine zipper motif was also proposed for residues 570–591 (blue), and although it forms the characteristic α-helix, it does not interact with the DNA in the dimer and is not part of the dimer interface. The previously predicted helix-turn-helix motif (residues 698–733) is colored in dark blue [77]. As our structure shows that the two helices are connected with a long flexible loop, helix-loop-helix motif is a more appropriate classification.

In addition to the well-characterized features of OBP described in previous paragraphs, we can address the structures and positioning of several other sequence-conserved elements. There appears to be an unexpected role for the conserved F553XXKYL motif: E555 forms a salt bridge with R607 of the partner monomer (Fig. 7A). This observation redefines our understanding of this motif's function. While previously implicated in DNA-binding domain folding [41] and temperature-sensitive DNA-binding (through K556A mutation studies [74], our structural data suggest its primary role is maintaining dimer integrity through inter-monomer contacts rather than directly participating in DNA recognition.

At the structure's apex, we identify an exposed flexible loop containing the PEST sequence (residues 265–282) (Fig. 7B). The positioning of this degradation-targeting motif [75, 76] in an accessible location has important implications for viral regulation and suggests a mechanism for controlled protein turnover during the viral replication cycle. Specifically, OBP has been shown to undergo efficient degradation by the ubiquitin-proteasome pathway in neuronal cells [78, 79], and OBP can be phosphorylated during a productive infectious cycle [80]. It is an intriguing possibility that the regulation of the initiation of HSV-1 replication by proteolytic degradation of OBP is a physiologically relevant event during latency and productive lytic infection.

Some conserved elements revealed surprising structural organizations. It has, for example, been proposed that residues 150–171 containing 4 regularly spaced leucines form a leucine zipper. However, rather than forming the predicted α-helical zipper structure with a hydrophobic interaction surface [81], this region adopts a mixed conformation comprising α-helix, β-strand, and random coil elements (Fig. 7C). In contrast, a second predicted pseudo-leucine zipper motif (residues 570–591), previously implicated in DNA binding [77], does form the characteristic α-helical structure but is positioned too far from both the DNA-binding surface and dimer interface to serve its proposed function (Fig. 7D).

We also observe a helix-loop-helix motif consisting of two helices connected by a loop (residues 698–733) that might challenge existing models of DNA recognition. This motif, previously predicted as a helix-turn-helix, has been proposed to mediate sequence-specific DNA binding [77], but our structure shows that it is positioned too far from the DNA in the dimer configuration (Fig. 7D). This motif may function during later stages of the unwinding process and engage with DNA after initial origin recognition has triggered conformational changes in the dimeric OBP complex.

### Multiple oligomeric states indicate dynamic assembly during origin activation

**Figure 8.**
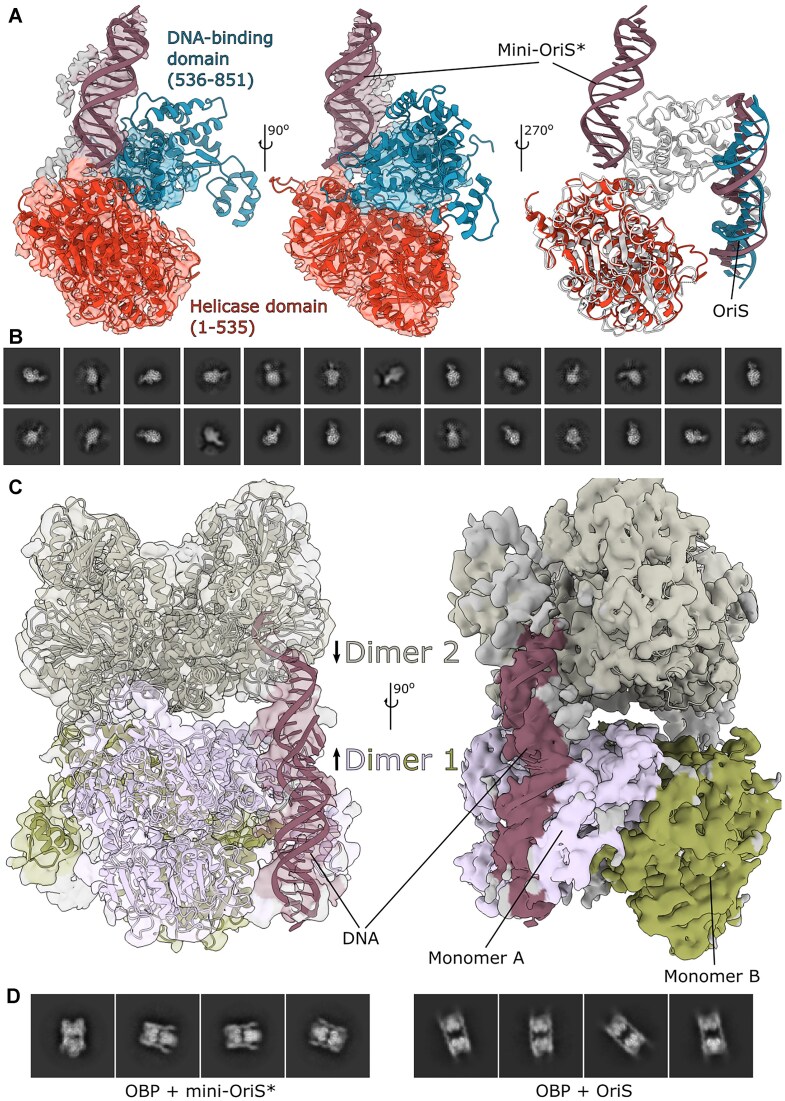
OBP monomeric and oligomeric states. (**A**) The structure of monomer A was rigid-body fitted into the monomer + mini-OriS* map. While the helicase domain (red) fits well into the map, the DNA-binding domain (blue) moves drastically, as seen by the poor fitting of the domain into the map. A modeled minimal OriS* could additionally be fitted into the map (purple), which reveals a dramatic reorganization compared to the dimer. On the right, an overlay between rigid-body fitted OBP monomer (red) + mini-OriS* (purple) and monomer A from OBP dimer (white) + OriS (purple and blue) shows the dramatic movement of the DNA between the two states. (**B)**. Representative 2D classes of the monomer, revealing preferred orientation. (**C)**. Cryo-EM map showing two dimers of OBP assembling in a head-to-head fashion in complex with mini-OriS*. The conformational change in OBP described above has not taken place. The two dimers have been fitted into the map (dimer 1 colored in plum and green, dimer 2 in gray). Two copies of mini-OriS* (purple) seem to be bound, one on each side of the dimer-dimer assembly. (**D)**. Representative 2D classes from two different datasets (Left: two dimers of OBP + mini-OriS*, with tight spacing between dimers with DNA bound along the sides; Right: two dimers of OBP + OriS, with larger spacing between dimers. Each dimer binds to two antiparallel OriS helices and the spacing between the dimers appears to be determined by the AT-rich spacer sequence.

To further investigate how single-stranded DNA influences OBP structure and function, we determined the cryo-EM structure of OBP in complex with a minimal OriS* bearing a single-stranded 3′ dT10 tail (mini-OriS*) [33]. Strikingly, OBP adopts a monomeric conformation in this complex, in contrast to the head-to-tail dimer observed with OriS DNA (Fig. 8A). Despite technical challenges from preferred particle orientation (Fig. 8B and Supplementary Fig. S9), the structure reveals dramatic conformational changes compared to the dimer state. Most notably, the mini-OriS* adopts an orientation almost perpendicular to the OriS position observed in the dimer. While the helicase domain and minimal OriS* are well-resolved, the limited resolution of the DNA-binding domain nevertheless indicates substantial domain rearrangement during the dimer-to-monomer transition. Similar large-scale domain swiveling have been reported in other helicases, such as eubacterial Rep and UvrD [82, 83]. It is of interest to note that the OBP + mini–OriS* complex is very stable and resists competition by a Box 1 duplex oligonucleotide for at least 20 min, whereas an OBP + OriS complex would dissociate within a few seconds [19]. The mini-OriS* DNA ligand is also an efficient activator of the ATPase activity of OBP, potentially due to disruption of an autoinhibitory interaction involving the *C*-terminal extension [41]. However, we emphasize that the exact DNA path and the orientation of the DNA-binding domain in the monomeric structure require further validation.

Our study also captured higher-order OBP assemblies, revealing two head-to-head dimers bound to putatively two copies of minimal OriS*, one along each side of the dimer-dimer assembly (Fig. 8C and D, left). At the current resolution, we cannot provide a detailed analysis of the DNA structure (Supplementary Fig. S10). Still, the two OBP dimers appear to retain the conventional dimer architecture without the domain rearrangement seen in the monomer. We also observed an additional assembly of OBP in the OBP + OriS dataset, albeit at a lower frequency. Here, each dimer appears to bind to two most likely antiparallel OriS helices. Each dimer thus appears to interact with Box 1 in one helix and with Box 2 in the second helix. The increased spacing between dimers as compared to the OBP + mini-OriS* dimer-dimer assembly would then be determined by the AT-rich spacer sequence (Fig. 8D, right). While the biological relevance of this arrangement is not yet clear, it demonstrates that OBP retains two active DNA-binding sites per dimer and is capable of forming noncanonical assemblies.

It is of importance to note that previous rotary-shadowcast electron microscopy studies of OBP + OriS complexes fixed with glutaraldehyde revealed a protein complex bound to a single copy of OriS, most likely consisting of two dimers binding cooperatively to the Box 1 and Box 2 arms of the OriS palindrome, forcing intervening DNA to bend significantly [84]. Furthermore, pre-unwinding complexes between OBP and OriS seem to be relatively stable to extreme dilution [85]. The existence of multiple distinct dimer-dimer assemblies suggests a dynamic assembly process where different oligomeric forms may serve specific functions during origin activation and DNA unwinding. These structures provide the first glimpse of how OBP might organize higher-order complexes at replication origins.

## Discussion

Our cryo-EM structures provide the first high-resolution view of HSV-1 OBP, revealing unexpected insights into viral DNA replication initiation. These structures, determined four decades after OBP’s initial discovery, refine our understanding of how HSV-1 replication origins are activated.

The high-resolution structures show a dimer binding to the high-affinity Box 1 site in OriS. The dimer exists in a head-to-tail configuration in which the *C*-terminal domain forms the primary dimer interface, contradicting previous models that suggested *N*-terminal dimerization [60]. These structures thus represent complex I previously identified through biochemistry [18, 84].

A particularly striking finding is the role of the *C*-terminus, containing the conserved ICP8-binding motif, as a sophisticated regulatory element. The *C*-terminus threads through the partner monomer near the ATP-binding pocket, creating an intricate regulatory mechanism that resolves a long-standing paradox: why *C*-terminal deletions enhance helicase activity but reduce overall replication efficiency [44]. This suggests a dual regulatory role – modulating both helicase activity and interactions with the essential partner protein ICP8. Our results provide the structural foundation for a regulatory mechanism that restricts OBP’s helicase activity to act on OriS and facilitates transfer of ICP8 to newly unwound DNA. Rather than merely serving as a structural element, the *C*-terminus emerges as a central coordinator of OBP’s diverse functions. In this aspect, it resembles the 2b domain of the UvrD helicase, a member of the SF1A helicase family, which regulates helicase activity by controlling dimer formation as well as allowing considerable conformational changes associated with function [83].

Our high-resolution structures also shine light on the molecular basis of origin recognition. The conserved RVKNL motif, particularly K758, makes precise base-specific contacts that explain how OBP differentially recognizes Boxes 1, 2, and 3 [40, 41]. Beyond these core interactions, we discovered an extensive network of peripheral contacts which may help to explain the dramatic differences in stabilities of complexes between OBP and OriS versus complexes between OBP and OriS*. The latter is, in this study, represented by a hairpin with a single-stranded tail [19, 20]. This elaborate recognition system likely ensures both specificity and stability during the early stages of replication initiation, as further discussed below.

From our cryo-EM reconstruction of an OBP monomer bound to the mini-OriS* DNA ligand, it is apparent that the transition from dimer to monomer involves considerable repositioning of the *C*-terminal domain, similar to the domain swiveling observed for Rep and UvrD helicases [82, 83]. A dimer-to-monomer transition is compatible with results from pull-down experiments demonstrating that OBP can simultaneously bind two duplex Box 1 oligonucleotides but only one copy of a hairpin with a single-stranded tail [33].

We also observe two distinct higher-order assemblies of OBP dimers in the presence of either the mini-OriS* ligand or full-length OriS, in a head-to-head manner. Importantly, our results show that both DNA-binding sites within each OBP dimer remain functional. However, we do not detect a stable complex of two OBP dimers simultaneously bound to a single OriS molecule, corresponding to the cross-linked complexes previously analyzed by electron microscopy [84, 85], implying that such a conformation may be transient and unstable prior to origin melting and helicase activation and therefore not possible to reconstruct using high-resolution cryo-EM.

**Figure 9.**
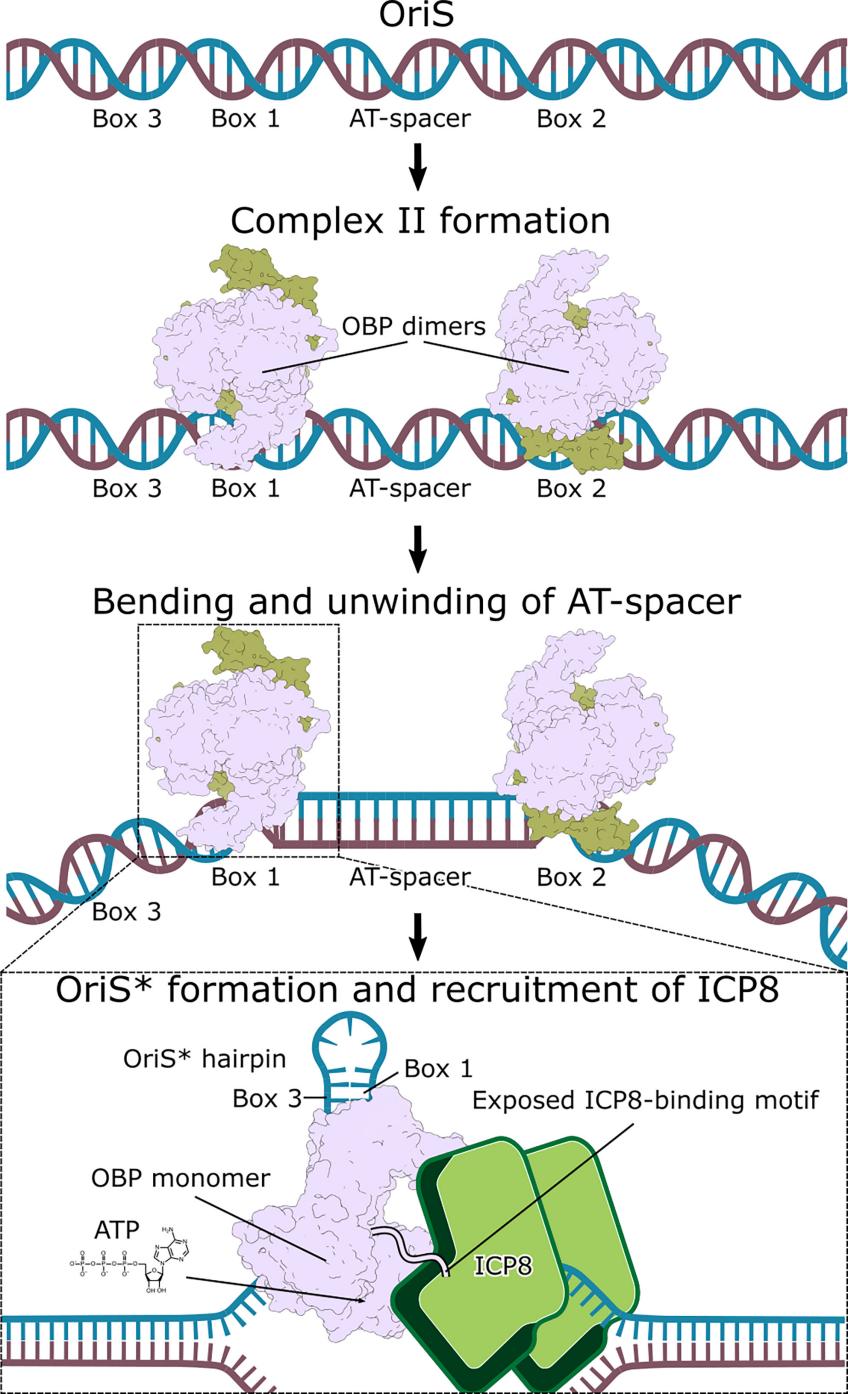
Conceptual figure displaying a possible mechanism for replication initiation for HSV-1 OriS. Two dimers bind cooperatively to Box 1 and Box 2 of a single copy of OriS, forming the so-called complex II. The dimer-dimer formation induces bending and destabilization of the AT-rich spacer sequence [18, 86]. The exposed single-stranded DNA allows for the binding of ICP8. Upon binding of ICP8, the *C*-terminus of OBP, containing the ICP8-binding motif, is released, which stimulates ATP hydrolysis. In turn, limited unwinding of minimal OriS occurs, leading to formation of the Box 3-Box 1 hairpin OriS* [17]. At some point during this sequence of events, OBP might undergo a dimer-to-monomer transition and form a more stable unwinding complex together with ICP8 [84, 85]. How the remaining OBP protomers of the dimer-dimer assembly are arranged after dimer-to-monomer transition is unknown. As the unwinding progresses, the single-stranded DNA gets decorated with ICP8 in a cooperative fashion [87].

To summarize, together with earlier biochemical and genetic data, our structural findings lend support to a model for activation of the HSV-1 OriS origin (Fig. 9). Two OBP dimers bind cooperatively to the Box 1 and Box 2 arms of OriS. When correctly positioned, as in a dimer-dimer assembly like complex II, the dimers may induce bending and destabilization of the AT-rich spacer [18, 86]. This distortion could promote transient exposure of single-stranded DNA, which facilitates ICP8 binding. ICP8 interaction may in turn release the autoinhibitory OBP *C*-terminal extension, leading to ATP hydrolysis and further conformational rearrangement in one or more OBP protomers. These transitions may initiate limited unwinding and favor formation of the Box 3–Box 1 hairpin structure (OriS*) [17]. OBP together with ICP8 then forms a more stable unwinding complex capable of further DNA strand separation, consistent with observations from electron microscopy studies [84, 85].

Since herpesviruses establish latent infections lasting for the lifetime of the infected individual, there is always the risk of reactivation of virus replication and re-emergence of clinical disease [6]. It has been argued that ubiquitin-dependent degradation of OBP might contribute to establishing and maintaining neuronal latency of HSV-1 [78, 79]. The identification of an exposed PEST sequence might offer an experimental route to further examine the physiological control of HSV-1 latency and reactivation.

Treatment of HSV-1 infections relies traditionally on inhibitors of the UL30 DNA polymerase. More recently, helicase-primase inhibitors have been developed that can control recurrent herpes disease and reduce the reactivation of latent infections [88]. Our high-resolution structures of OBP in complex with DNA ligands suggest a number of novel target sites suitable for drug development. We would specifically like to highlight the unique DNA-binding motif, the interactions responsible for dimer formation and interaction with the single-strand DNA binding protein ICP8, as well as exploring the unusual configuration of the ATP-binding pocket as observed in our OBP-dimer structure. These diverse targeting options could help overcome the growing challenge of resistance to current polymerase-targeting antivirals by providing entirely new mechanisms of action.

In conclusion, our work represents a significant advance in understanding both fundamental mechanisms of HSV-1 replication initiation and the potential for therapeutic intervention. The complex structural transitions and regulatory mechanisms we observe illuminate crucial aspects of viral biology and identify promising avenues for developing next-generation antiviral treatments. These insights provide a critical foundation for combating drug-resistant HSV-1 infections and possibly extending antiviral treatment to prevent secondary disease following herpesvirus infections.

## Supplementary Material

gkaf1029_Supplemental_File

## Data Availability

The coordinates have been deposited in the Protein Data Bank (PDB) with accession codes PDB-ID: **9HGI** (OBP + OriS-6AT) and **9HGJ** (OBP + OriS + ATPγS). The cryo-EM maps have been deposited in the Electron Microscopy Data Bank (EMDB) with accession codes **EMD-52135** (Consensus map of OBP + OriS-6AT), **EMD-52136** (Local refinement map of OBP + OriS-6AT monomer A), **EMD-52137** (Local refinement map of OBP + OriS-6AT monomer B), **EMD-52145** (Refinement map of OBP + OriS + ATPγS), **EMD-52146** (Composite map of dimer-dimer assembly OBP + mini-OriS*), **EMD-52147** (Consensus map of dimer-dimer assembly OBP + mini-OriS*), **EMD-52148** (Local refinement map of dimer-dimer assembly OBP dimer 1), **EMD-52149** (Local refinement map of dimer-dimer assembly OBP dimer 2), **EMD-52150** (Map of monomeric state OBP + mini-OriS*). The raw cryo-EM movies have been deposited in the Electron Microscopy Public Image Archive (EMPIAR) with accession codes **EMPIAR-12878** (OBP + mini-OriS*), **EMPIAR-12879** (OBP + OriS + ATPγS), and **EMPIAR-12880** (OBP + OriS-6AT).
