# Supplementary material for: The herpes simplex origin-binding protein: mechanisms for sequence-specific DNA binding and dimerization revealed by Cryo-EM
Source: Nucleic Acids Res. 2025 Oct 23;53(19):gkaf1029. doi: 10.1093/nar/gkaf1029 (PMC12543377; doi:10.1093/nar/gkaf1029)
Supplement: gkaf1029_Supplemental_File [file gkaf1029_supplemental_file.pdf]

Supplementary Information -
The Herpes simplex Origin Binding Protein: Mechanisms for sequence specific DNA binding and dimerization revealed by Cryo-EM

Emil Gustavsson[1,2], Kay Grünewald[2,3,4], Per Elias[5] and B. Martin Hällberg[1,2]*

[1] Department of Cell and Molecular Biology, Karolinska Institutet, 171 77 Stockholm, Sweden

[2] CSSB Centre for Structural Systems Biology, Deutsches Elektronen-Synchrotron DESY, Notkestraße 85, 22607 Hamburg, Germany

[3] Leibniz-Institute of Virology (LIV), Martinistraße 52, 20251 Hamburg, Germany

[4] Department of Chemistry, University of Hamburg, Martin-Luther-King-Platz 6, 20146 Hamburg, Germany

[5] Institute of Biomedicine, Department of Medical Biochemistry and Cell Biology, Sahlgrenska Academy, University of Gothenburg, Box 440, 405 30 Gothenburg, Sweden

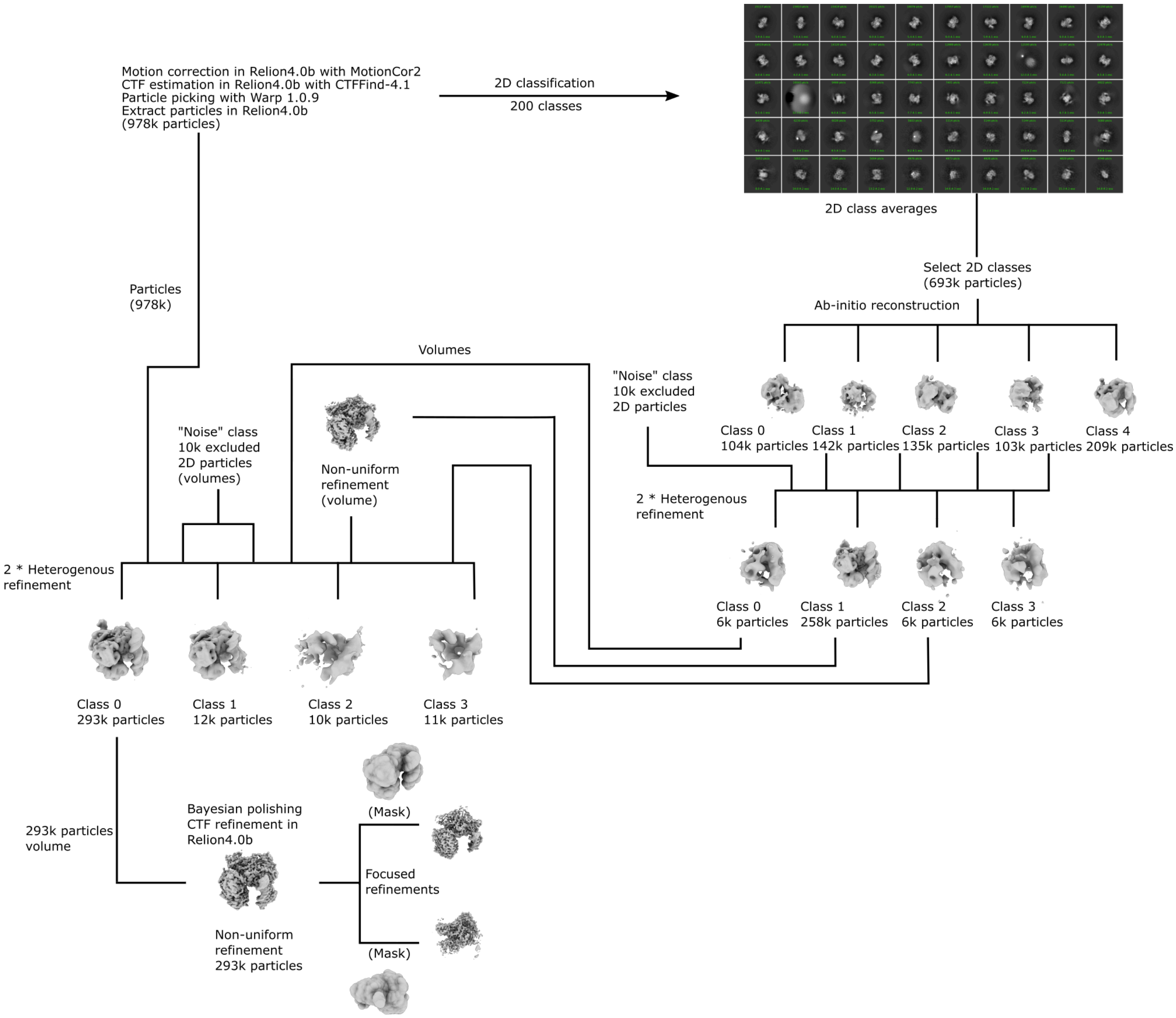

Supplementary Figure 1. Data processing scheme of OBP + OriS-6AT. Motion correction and CTF estimation were performed in Relion 4.0b, with MotionCor2 and CTFFind 4.1, respectively[1–3]. Particle picking was performed with Warp 1.0.9[4], and extraction was performed in Relion 4.0b. The subsequent steps were performed in CryoSPARC v.3.2.0[5], with the exception of Bayesian polishing and CTF refinement, which were done in Relion 4.0b.

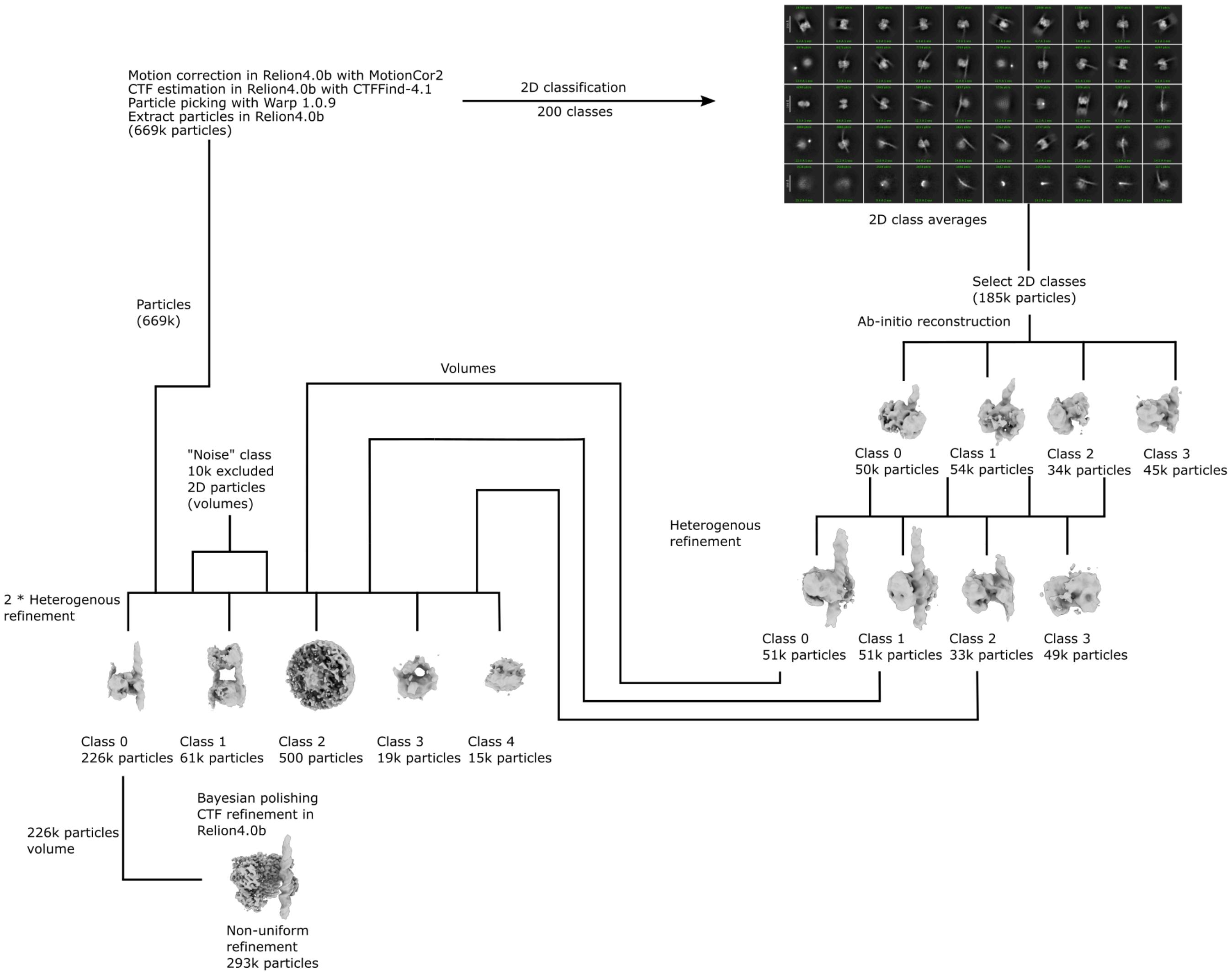

Supplementary Figure 2. Data processing scheme of OBP + OriS + ATPγS. Motion correction and CTF estimation were performed in Relion 4.0b, with MotionCor2 and CTFFind 4.1, respectively[1–3]. Particle picking was performed with Warp 1.0.9[4], and extraction was performed in Relion 4.0b. The subsequent steps were performed in CryoSPARC v.3.2.0[5], with the exception of Bayesian polishing and CTF refinement, which were done in Relion 4.0b.

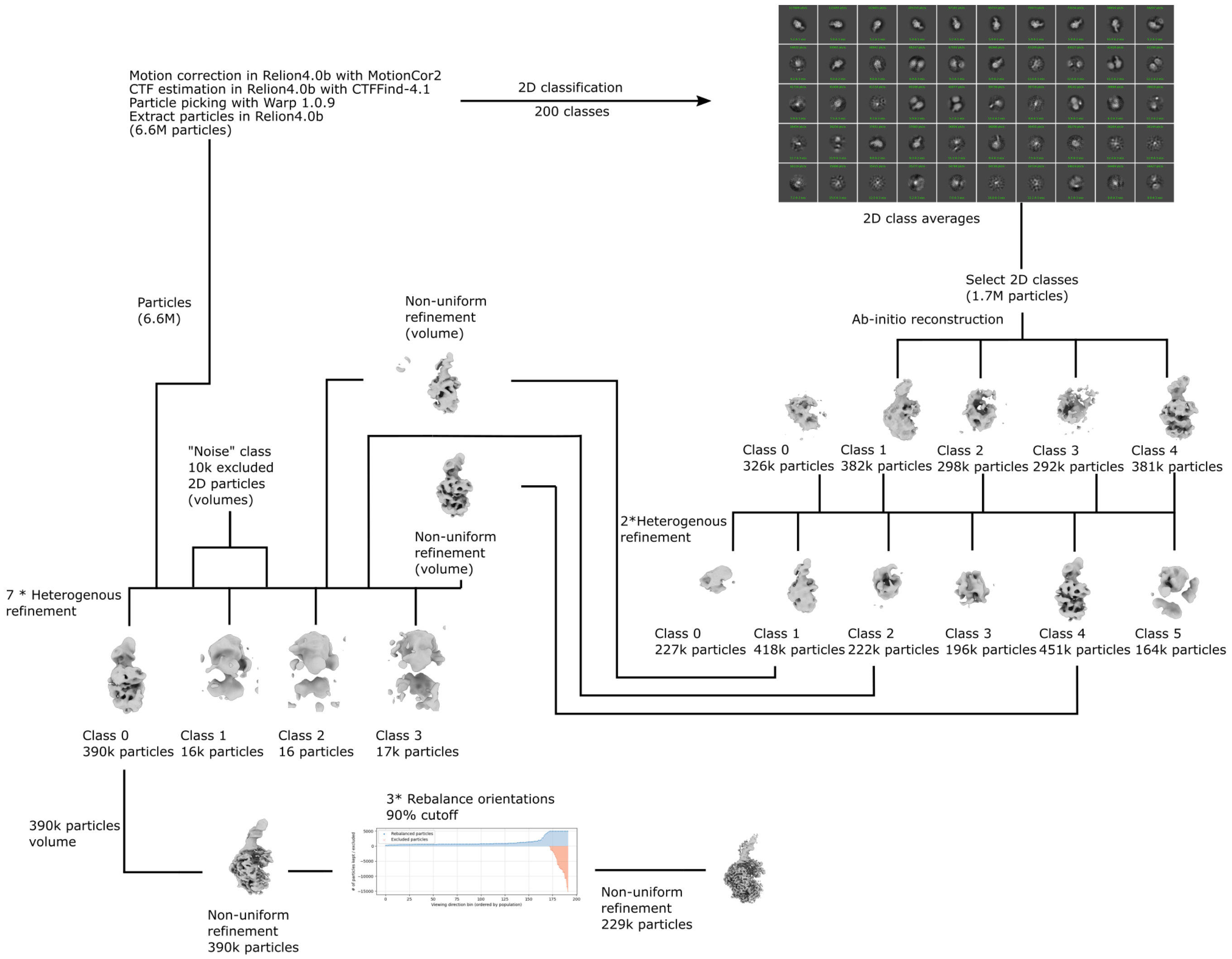

Supplementary Figure 3. Data processing scheme of monomeric OBP + mini-OriS*. Motion correction and CTF estimation were performed in Relion 4.0b, with MotionCor2 and CTFFind 4.1, respectively[1–3]. Particle picking was performed with Warp 1.0.9[4], and extraction was performed in Relion 4.0b. The subsequent steps were performed in CryoSPARC v.3.2.0[5].

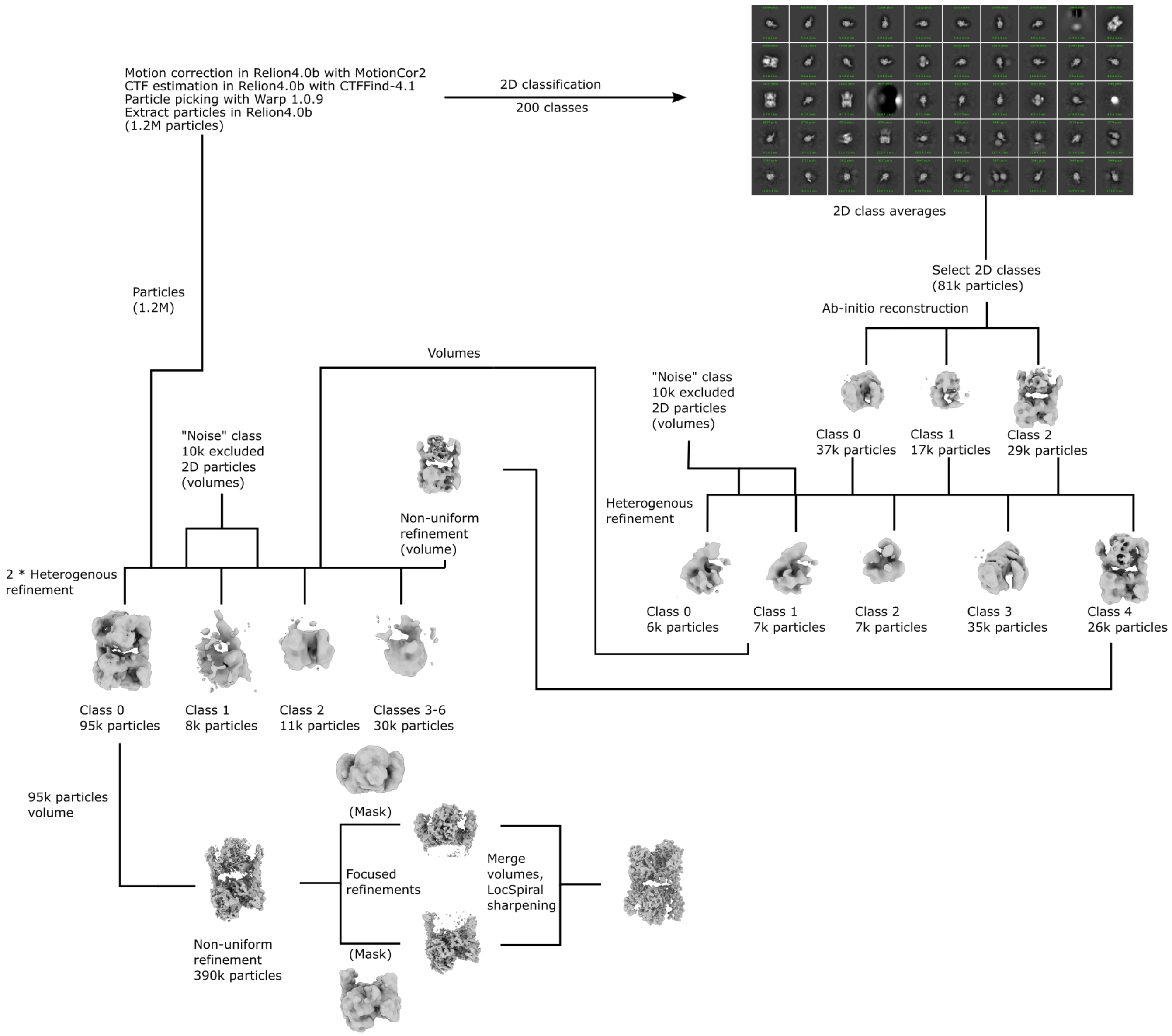

Supplementary Figure 4. Data processing scheme of OBP + mini-OriS* dimer-dimer assembly. Motion correction and CTF estimation were performed in Relion 4.0b, with MotionCor2 and CTFFind 4.1, respectively[1–3]. Particle picking was performed with Warp 1.0.9[4], and extraction was performed in Relion 4.0b. The subsequent steps were performed in CryoSPARC v.3.2.0[5].

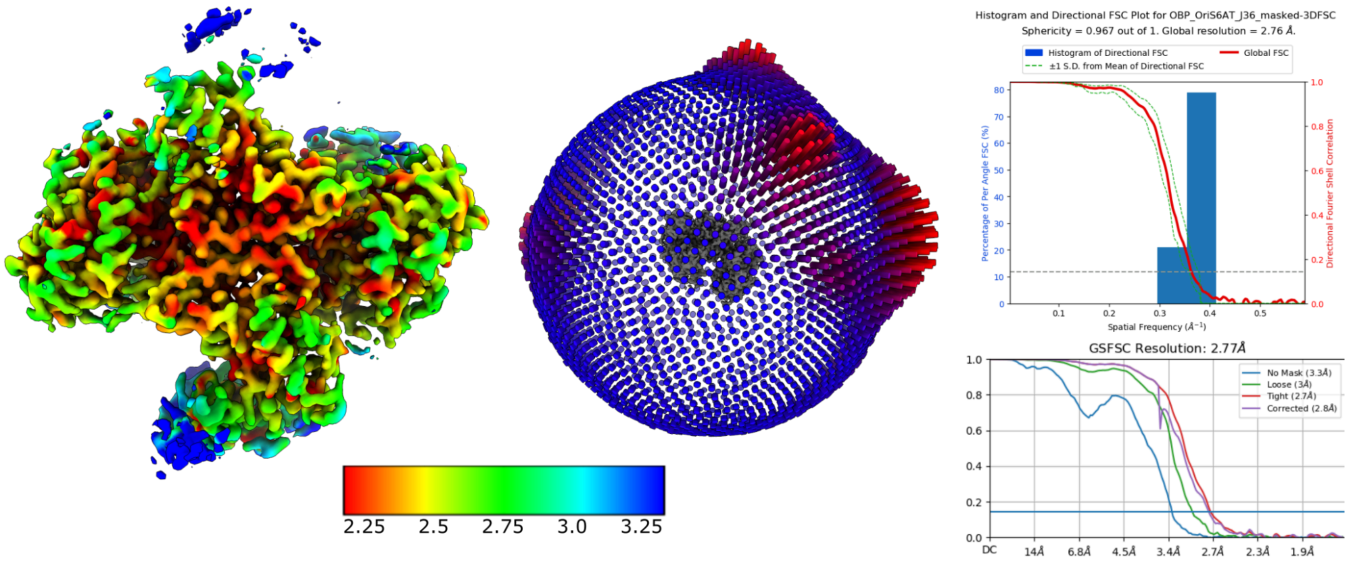

Supplementary Figure 5. Cryo-EM reconstruction post-processed by DeepEMhancer of the dimeric OBP + OriS-6AT colored by local resolution determined by local resolution estimation of the refinement map in cryoSPARC [5,6]. The OBP core reaches a resolution of 2.77Å, whereas the more peripheral regions, like the DNA-binding domain and DNA, are in the range of 3-4Å. There is a slightly preferred orientation, although not detrimental to the map reconstruction.

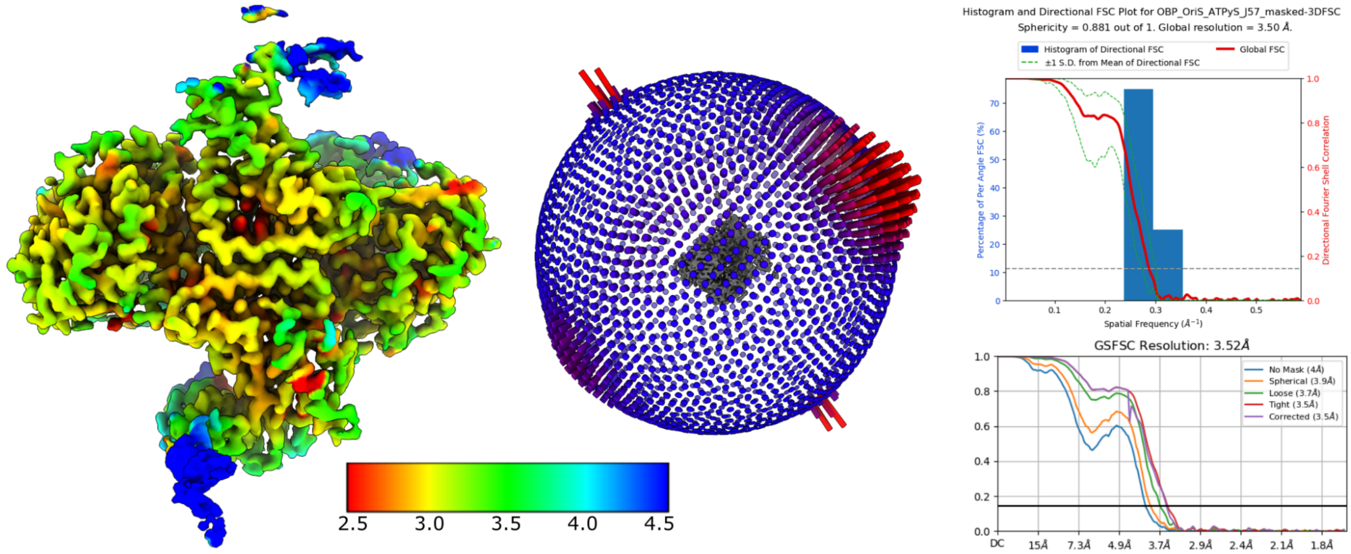

Supplementary Figure 6. Cryo-EM reconstruction post-processed by DeepEMhancer of the dimeric OBP + OriS + ATPγS colored by local resolution determined by local resolution estimation of the refinement map in cryoSPARC [5,6]. The OBP core reaches a resolution of 3Å, whereas the more peripheral regions, like the DNA-binding domain and DNA, are in the range of 3.5-4Å. There is a slightly preferred orientation, although not detrimental to the map reconstruction.

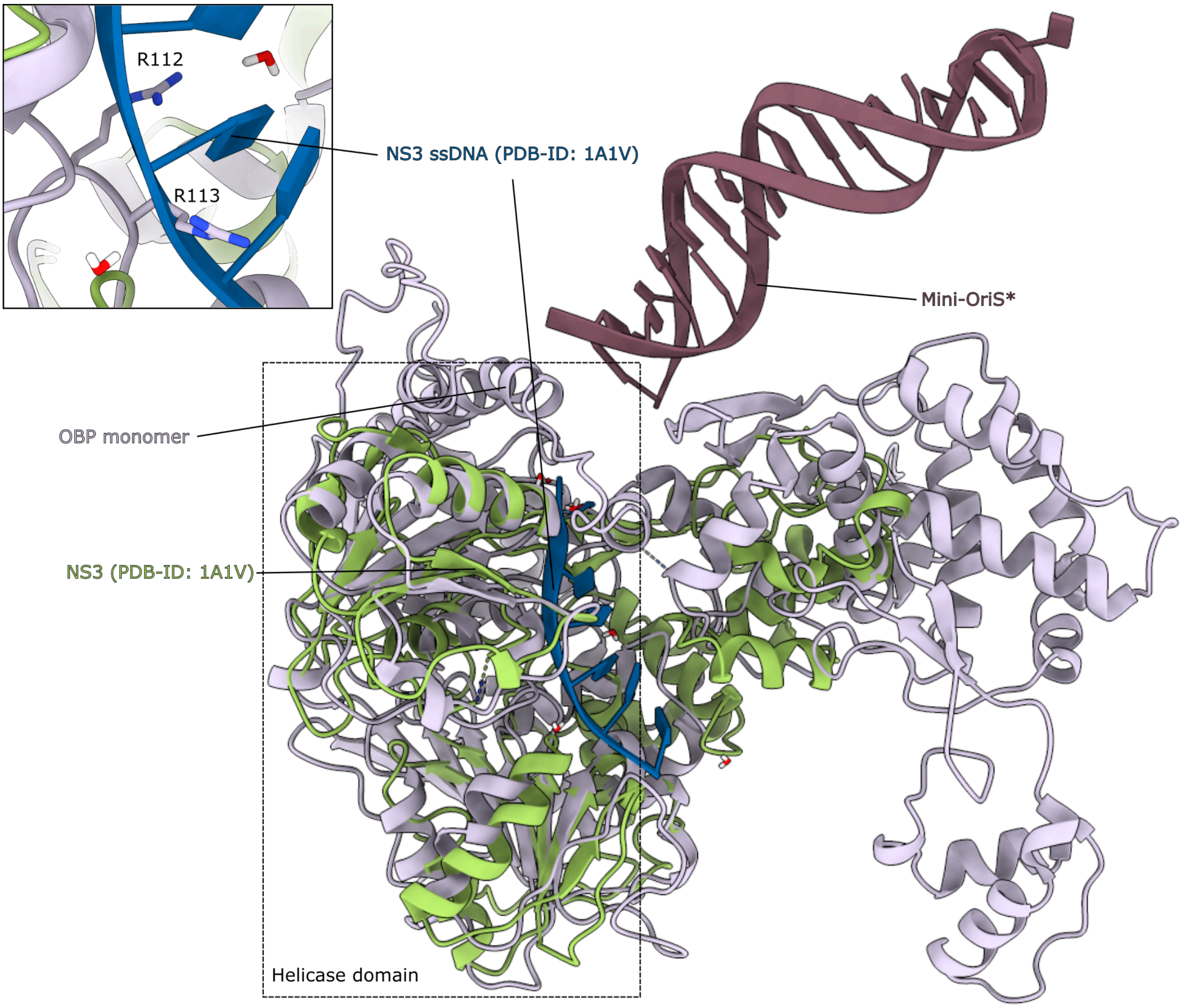

Supplementary Figure 7. OBP overlayed with another SF2 helicase, the Hepatitis C virus NS3 (PDB-ID: 1A1V)**.** The helicase domains show remarkable similarities, and the NS3 structure might give indications for OBP-ssDNA interactions. Positioning of R112 and R113 explains previous observations that R112A and R113A mutations abolish ssDNA-binding while maintaining ATPase activity (inset)[7].

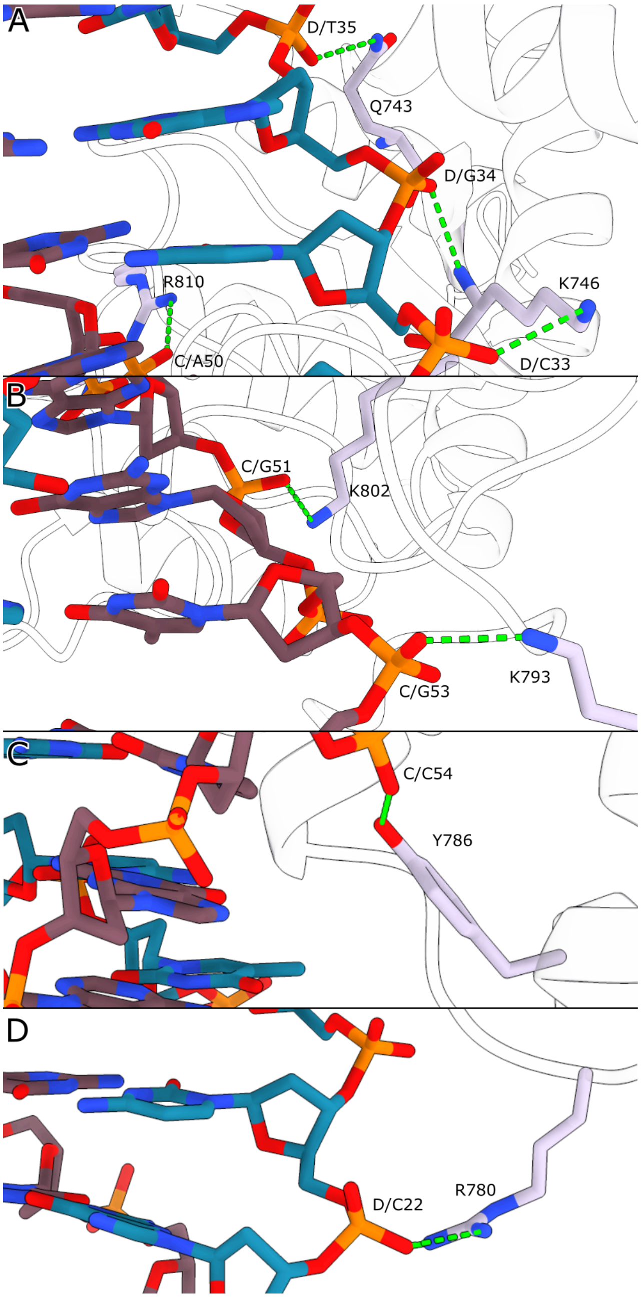

Supplementary Figure 8. Hydrogen bonding network in OBP + OriS-6AT. **A.** Q743 hydrogen bonds with the phosphate group in D/T35. K746 side chain bonds with the D/C33 phosphate, while the main chain amide hydrogen bonds with D/G34 phosphate. R810 forms a hydrogen bond with C/A50. **B.** K793 hydrogen bonds with C/G53 and K802 hydrogen bonds with C/G51. **C.** Y786 hydrogen bonds with C/C54. **D.** R780 hydrogen bonds with D/C22.

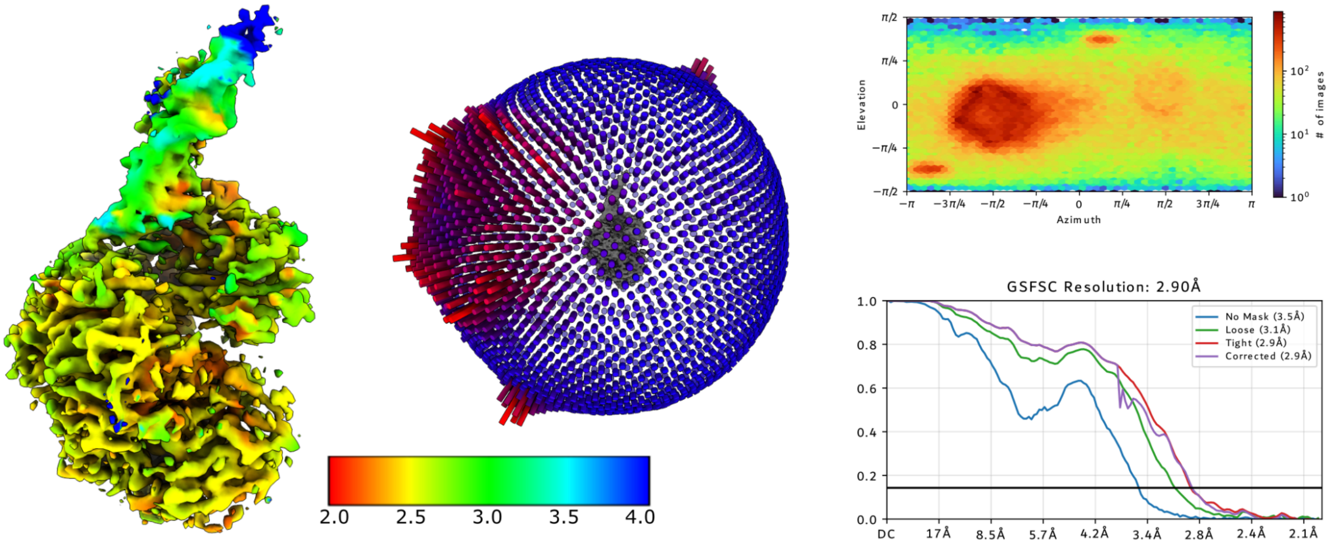

Supplementary Figure 9. Cryo-EM reconstruction of the monomeric OBP + mini-OriS* colored by local resolution determined by local resolution estimation of the refinement map in cryoSPARC [5]. The resolution is unreliable due to severe preferred orientation problems.

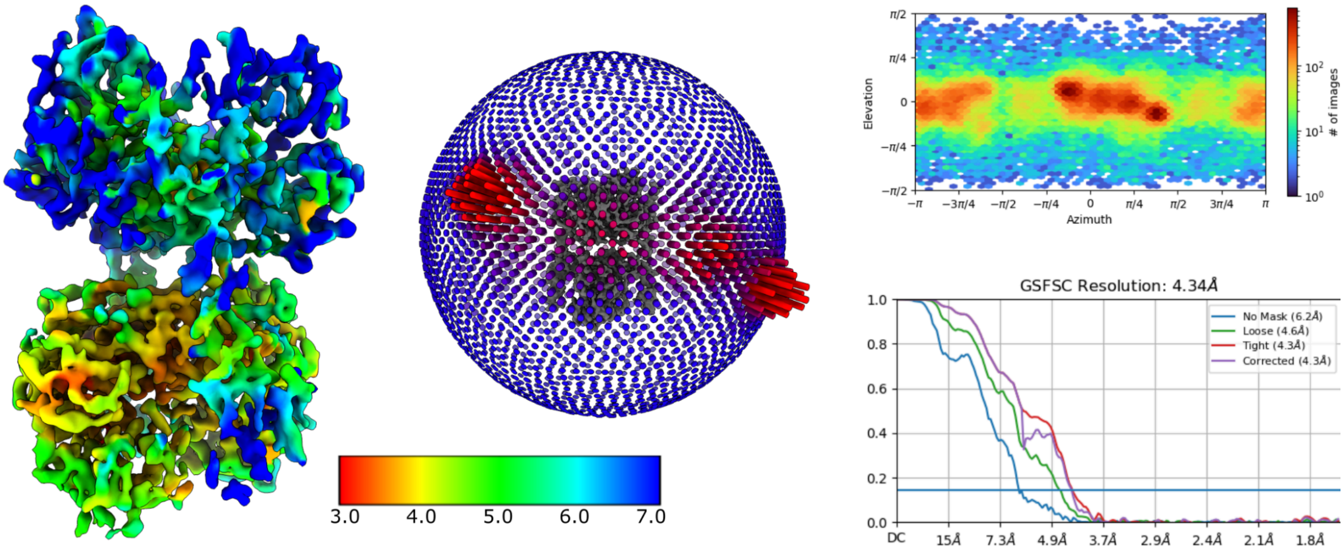

Supplementary Figure 10. Cryo-EM reconstruction post-processed by LocSpiral of the dimer-dimer assembly of OBP + mini-OriS* colored by local resolution determined by local resolution estimation of the refinement map in cryoSPARC [5,8].

Supplementary Table 1. Cryo-EM data collection, refinement and validation statistics. This table provides the parameters and statistics for the data collection, processing, refinement, and structure validation of the cryo-EM structures. Refinement statistics were generated using the Servalcat package.

	**OBP + OriS-6AT**	**OBP + OriS + ATPγS**	**OBP + mini-OriS* (dimer-dimer assembly)**	**OBP + mini-OriS* (monomer)**
**PDB code**	9HGI	9HGJ	-	-
**EMDB code**	Consensus map: EMD-52135, Monomer A: EMD-52136, Monomer B: EMD-52137	Consensus map: EMD-52145	Composite map: EMD-52146, Consensus map: EMD-52147, Dimer 1: EMD-52148, Dimer 2: EMD-52149	Consensus map: EMD-52150
**Data collection and processing**				
Microscope/detector	Krios G3/K3	Krios G3/K3	Krios G3/K3	Krios G3i X-FEG/K3
Magnification	105kx	105kx	105kx	165kx
Voltage	300kV	300kV	300kV	300kV
Electron exposure (e-/Å^2)	50	54	50	58
Defocus range (μm)	-0.75 to -2	-0.75 to -2	-0.75 to -2	-0.5 to -1.5
Pixel size (Å)	0.85	0.85	0.85	0.505
Symmetry imposed	C1	C1	C1	C1
Final particle images (*n*)	293067	226401	95006	228539
Map resolution (Å) ($FSC_{0.143}$)	2.77	3.53	4.34	2.9
Map sharpening B factor (Å^2)	Local Filter	Local Filter	Local Filter	Local Filter
**Refinement**				
Initial model	AlphaFold2 + ModelAngelo model	Our OBP + OriS-6AT model	Our OBP + OriS-6AT model	Our OBP + OriS-6AT model
**Model composition**				
Non-hydrogen atoms	13657	13588	-	-
Protein residues	1623	1608	-	-

Nucleotide residues	48	48	-	-
Water	-	-	-	-
Other	-	1 Mg^{2+}<br>2 ATPγS	-	-
**R.m.s. deviations (RMSZ)**				
Bond lengths	0.53	0.52	-	-
Bond angles	0.93	0.98	-	-
**Validation**				
MolProbity score	1.18	0.93	-	-
Clashscore	3.09	0.15	-	-
Poor rotamers (%)	0	0.29	-	-
**Ramachandran plot**				
Favored (%)	98	95	-	-
Allowed (%)	2	5	-	-
Disallowed (%)	0	0	-	-

Supplementary references

1. Zivanov, J., Nakane, T. & Scheres, S. H. W. Estimation of high-order aberrations and anisotropic magnification from cryo-EM data sets in *RELION* -3.1. *IUCrJ* **7**, 253–267 (2020).
2. Zheng, S. Q. *et al.* MotionCor2: anisotropic correction of beam-induced motion for improved cryo-electron microscopy. *Nat. Methods* **14**, 331–332 (2017).
3. Rohou, A. & Grigorieff, N. CTFFIND4: Fast and accurate defocus estimation from electron micrographs. *J. Struct. Biol.* **192**, 216–221 (2015).
4. Tegunov, D. & Cramer, P. Real-time cryo-electron microscopy data preprocessing with Warp. *Nat. Methods* **16**, 1146–1152 (2019).
5. Punjani, A., Rubinstein, J. L., Fleet, D. J. & Brubaker, M. A. cryoSPARC: algorithms for rapid unsupervised cryo-EM structure determination. *Nat. Methods* **14**, 290–296 (2017).
6. Sanchez-Garcia, R. *et al.* DeepEMhancer: a deep learning solution for cryo-EM volume post-processing. *Commun. Biol.* **4**, 874 (2021).
7. Marintcheva, B. & Weller, S. K. Helicase Motif Ia Is Involved in Single-Strand DNA-Binding and Helicase Activities of the Herpes Simplex Virus Type 1 Origin-Binding Protein, UL9. *J. Virol.* **77**, 2477–2488 (2003).
8. Kaur, S. *et al.* Local computational methods to improve the interpretability and analysis of cryo-EM maps. *Nat. Commun.* **12**, 1240 (2021).
